# ﻿Additions to woody litter fungi of *Byssosphaeria*, *Phaeoseptum* and *Pseudothyridariella* (Pleosporales, Ascomycota) from China

**DOI:** 10.3897/mycokeys.122.161224

**Published:** 2025-09-05

**Authors:** Wen-Xin Su, Ranagul Tieliwadi, Wen-Ying Su, Xiao Li, Rong Xu

**Affiliations:** 1 Joint International Research Laboratory of Modern Agricultural Technology, Ministry of Education, Jilin Agricultural University, Changchun, 130118, China Jilin Agricultural University Changchun China; 2 Lianyungang Academy of Agricultural Sciences, Lianyungang, 222006, China Lianyungang Academy of Agricultural Sciences Lianyungang China; 3 School of Food Science and Engineering, Yangzhou University, Yangzhou, 225127, China Yangzhou University Yangzhou China

**Keywords:** Molecular phylogeny, new host records, new species, pleosporalean fungi, taxonomy

## Abstract

Pleosporales, the largest order within Ascomycota, is globally distributed and exhibits remarkable host diversity. Fagaceae, a plant family of significant ecological importance in the Northern Hemisphere, harbors a wide array of fungal species. During an investigation of pleosporalean fungi associated with woody litter from Fagaceae plants, dead wood samples with fungal fruiting bodies were collected in Henan and Jiangxi provinces, China. In this study, we describe two novel species, *Pseudothyridariella fagacearum* and *Phaeoseptum biyangense*, and report a new host record for *Byssosphaeria siamensis*. These taxa are formally characterized using an integrative taxonomic approach, which combines detailed morphological characterization with maximum likelihood (ML) and Bayesian inference (BI) phylogenetic analyses of multi-locus sequence data (ITS, LSU, SSU, *rpb*2, and *tef*1-α). The newly designated species can be morphologically distinguished from other members of the respective genera based on the size of the asci and ascospores. This comprehensive study enhances our understanding of microfungi associated with Fagaceae, contributing to the knowledge of China’s biodiversity.

## ﻿Introduction

Wood litter fungi play a crucial role in forest ecosystems, secreting extracellular enzymes that break down complex organic substances such as lignin, cellulose, and hemicellulose, thereby facilitating the decomposition of organic matter and nutrient cycling (Krishna and Mohan 2017; Bani et al. 2018; Yu 2021). Some fungal taxa have a close association with specific host diseases and preferences, while most possess a wide distribution range and a diverse range of hosts. Therefore, current research on woody fungi has focused more on those that cause diseases in specific host plants. Beyond the key role in ecological systems, these fungi also exhibit potential for applications in medicinal properties, environmental pollution control, and the fermentation industry (Hu et al. 2021; Ingersoll 2023). For example, lignocellulosic waste and nitrogen-rich organic substrates can be co-digested through high-temperature anaerobic digestion with thermophilic fungi to produce biomethane (Ingersoll 2023).

Pleosporales is the largest and most diverse order within the class Dothideomycetes of the phylum Ascomycota, comprising more than 90 families and 1000 genera (Wijayawardene et al. 2022; Yang et al. 2022; Hyde et al. 2024). Within this order, the taxa predominantly function as saprobes, pathogens, endophytes, epiphytes, and/or parasites across various habitats (Põlme et al. 2020). In recent years, due to the increasing number of fungi discovered within Pleosporales, records of many new woody litter pleosporalean species residing in terrestrial or aquatic habitats have gradually increased in China (Zhang et al. 2009; Su et al. 2016, 2018, 2022; Huang et al. 2018; Jiang et al. 2019; Wanasinghe et al. 2020; Ren et al. 2022; Xie et al. 2022; Xu et al. 2022; Xiong et al. 2023).

Fagaceae is a species-rich plant family comprising nearly 1,000 species, including *Castanea* spp., *Cyclobalanopsis* spp., *Fagus* spp., and *Quercus* spp., mainly distributed in the Northern Hemisphere, with only a few species distributed in the Southern Hemisphere (Manos et al. 2001). Fagaceae species have high ecological value and are usually used as shade, street, or ornamental trees. Furthermore, this family is not only used for timber production but also serves as an excellent source of soil and water conservation (Wu and Raven 1999). In addition, their fruits, wood, bark (cork), and the ecosystem services also hold significant economic value (Petit et al. 2013). As one of the most thoroughly studied plant families worldwide, its demonstrated value has driven significant applied arboriculture research. Researchers have utilized Fagaceae trees as models to study breeding, ecology, evolution, and genomics (Petit et al. 2013; Cannon et al. 2018; Zhou et al. 2022). To date, numerous fungal species have been reported in association with Fagaceae, including ectomycorrhizal (EM) fungi, soil fungi, and basidiomycetes (Wilson et al. 2012; Wu et al. 2018; He et al. 2022; Brown et al. 2023; Li et al. 2024; Niu et al. 2024). Notably, some of these fungi act as pathogens, causing leaf lesions and branch cankers in Fagaceae species (Gong et al. 2017; Qiu et al. 2017; Jiang and Tian 2019; Jiang et al. 2019, 2020, 2021a, b).

Jiangxi Province, located in southeastern China, covers a total area of 166,900 square kilometers, about 59% of which is vast forest (Yang et al. 2022). The mountainous terrain and large forest coverage of Jiangxi have made it historically one of the wildest places in China (Wu et al. 2018). Due to its abundant water resources, it is also known as one of China’s largest grain producers. The subtropical monsoon-type climate and sufficient moisture make Jiangxi a favorable region for fungal growth (Hu et al. 2022). Henan Province, situated in the central part of China, lies within the middle and lower reaches of the Yellow River and boasts well-developed river systems. It covers an area of over 167,000 square kilometers, about 25.47% of which is forest. The natural vegetation of Henan consists of deciduous forest and woodland over the plains, as well as deciduous and coniferous forests in the western highlands (Huang et al. 2019). Henan has a predominantly temperate climate with four distinct seasons (Gao 2024). The superior ecological environment is characterized by a rich diversity of fungi (Hu et al. 2022; Gao 2024). However, fungal investigations in these two regions primarily focus on macrofungi and freshwater fungi, while saprophytic fungi are often overlooked (Chen 2022; Xu and Hu 2023).

This study aimed to describe two new species of wood litter fungi, *Phaeoseptum biyangense* and *Pseudothyridariella fagacearum*, and to report a new record of *Byssosphaeria siamensis*, based on morphological and multi-locus phylogenetic analyses. The new taxa and known species are provided with detailed illustrations and morphological descriptions. These data contribute to the increasing number of ascomycetous fungi known from China and enrich the diversity of saprobic fungi associated with Fagaceae.

## ﻿Materials and methods

### ﻿Sample collection and isolation

Plant litter specimens were collected from Ji’an City, Jiangxi Province (27°4′–27°36'N, 114°–114°47'E) and Zhumadian City, Henan Province (32°40'25.49"N, 113°28'33.66"E). The specimens were stored in sealed Ziploc bags, along with details such as the collection location, time, and host (Rathnayaka et al. 2025). Fungal isolation was performed according to Senanayake et al. (2020). Single spores were isolated on potato dextrose agar (PDA) and incubated at 25 °C in the dark for 24 h (Monkai et al. 2021). Germinated spores were aseptically transferred to new PDA plates and incubated at 25 °C for 2 weeks. Pure cultures were deposited at the Engineering Research Center of Edible and Medicinal Fungi, Ministry of Education, Jilin Agricultural University (CCMJ), Changchun, China, and type specimens were deposited at the Engineering Research Center of Edible and Medicinal Fungi, Ministry of Education culture collection (EMFCC), Changchun, China. New taxa were registered in MycoBank (https://www.mycobank.org/).

### ﻿Morphological observation

The samples were examined using a dissecting microscope (Zeiss Stemi 2000C, Carl Zeiss AG, Germany) equipped with a digital camera. After locating the ascomata under a Leica DFC450C microscope (Leica, Heidelberg, Germany), sections were carefully cut using a sterilized scalpel, then mounted on glass slides with distilled water for microscopic observation. The structure and size of ascomata, peridium, ostiole, hamathecium, asci, and ascospores were observed and photographed using a Zeiss AX10 compound microscope (Carl Zeiss AG, Germany) with an Axiocam 506 digital camera under differential interference contrast (DIC) illumination. The ZEN 3.3 (blue edition) was used for microscopic measurements (ZEISS, Germany). Images were subsequently processed using Adobe Photoshop CC2020 software (Adobe Systems Inc., San Jose, CA, USA).

### ﻿DNA extraction, PCR amplification, and sequencing

Genomic DNA was extracted from pure cultures using the NuClean PlantGen DNA Kit (CWBIO, China) according to the manufacturer’s protocol. The internal transcribed spacer region of ribosomal DNA (ITS) (White et al. 1990), the large subunit (LSU) of ribosomal DNA (Vilgalys and Hester 1990), the RNA polymerase II second-largest subunit (*rpb*2) (Voglmayr et al. 2016), and the translation elongation factor 1-alpha (*tef*1-α) (Rehne and Buckley 2005) were amplified. The PCR amplifying conditions of ITS, LSU, and SSU are the same: an initial denaturation step of 5 min at 94 °C, followed by 35 cycles of 30 s at 94 °C, 30 s at 53 °C, and 90 s at 72 °C, and a final extension step of 10 min at 72 °C, and 4 °C for holding temperature. For *rpb*2 and *tef*1-α, an initial denaturation step of 5 min at 94 °C was performed, followed by 35 cycles of 30 s at 94 °C, 45 s at 56 °C (*rpb*2) or 60 s at 52 °C (*tef*1-α), and 90 s at 72 °C, with a final extension step of 10 min at 72 °C and a holding temperature of 4 °C.

The amplification reactions were performed in 20 μL PCR mixtures containing the following components: 9 μL of ddH_2_O, 10 μL of 2× EsTaq MasterMix (Dye), 0.4 μL of DNA template, and 2 μL of each primer (2 μmol/μL). The PCR products were analyzed by electrophoresis on a 1% agarose gel for visualization. Subsequently, the PCR products were sent to Sangon Biotech (Shanghai) Co., Ltd., China, for sequencing.

### ﻿Phylogenetic analysis

The nucleotide sequences used for phylogenetic analyses in the present investigation were systematically selected by searching the GenBank database (www.ncbi.nlm.nih.gov/BLAST) using BLAST (Suppl. material 1: table S1). The alignments for each locus were generated using MAFFT v. 7.0 (http://mafft.cbrc.jp/alignment/server/) (Katoh et al. 2019), and ambiguous nucleotides were manually adjusted in AliView (Katoh et al. 2019). Nucleotide sequence alignments of individual loci were concatenated using SequenceMatrix v1.7.8 (Vaidya et al. 2011) to assemble the multi-locus phylogenetic dataset.

Phylogenetic analyses were conducted using maximum likelihood (ML) and Bayesian inference (BI) methods. Maximum likelihood analyses were executed using RAxML-RAxMLHPC2 v.8 on XSEDE (8.2.12) (Stamatakis 2006; Stamatakis et al. 2008) via the CIPRES web portal (http://www.phylo.org/portal2/). The phylogenetic tree was constructed using 1,000 non-parametric bootstrap replicates, with the best-scoring tree determined through a comparative evaluation of likelihood scores among alternative trees generated from individual runs, utilizing the GTRGAMMA nucleotide substitution model. Nodes with ML bootstrap values of 70% or higher were annotated accordingly. BI analyses were implemented using MrBayes v. 3.2.6 (Ronquist et al. 2003). The program executed four chains simultaneously for 3,000,000 iterations per chain, and the tree was sampled once every 100^th^ generation. The initial 20% of the sampled data was discarded as a burn-in period. At the same time, the remaining 8,000 trees were used to calculate the posterior probabilities for the majority-rule consensus tree (the critical value for the topological convergence diagnosis was set at 0.01) (Cai et al. 2006). Bayesian posterior probabilities (BPP) equal to or greater than 0.90 were marked at each node.

The phylogenetic tree was visualized using FigTree v. 1.4.4 (Rambaut 2018) and subsequently refined for publication in Adobe Illustrator CS6 (Adobe Systems, USA). All newly generated sequences from this study have been deposited in GenBank.

## ﻿Results

### ﻿Phylogenetic analyses

Phylogenetic analyses of *Pseudothyridariella*

**Figure 1.**
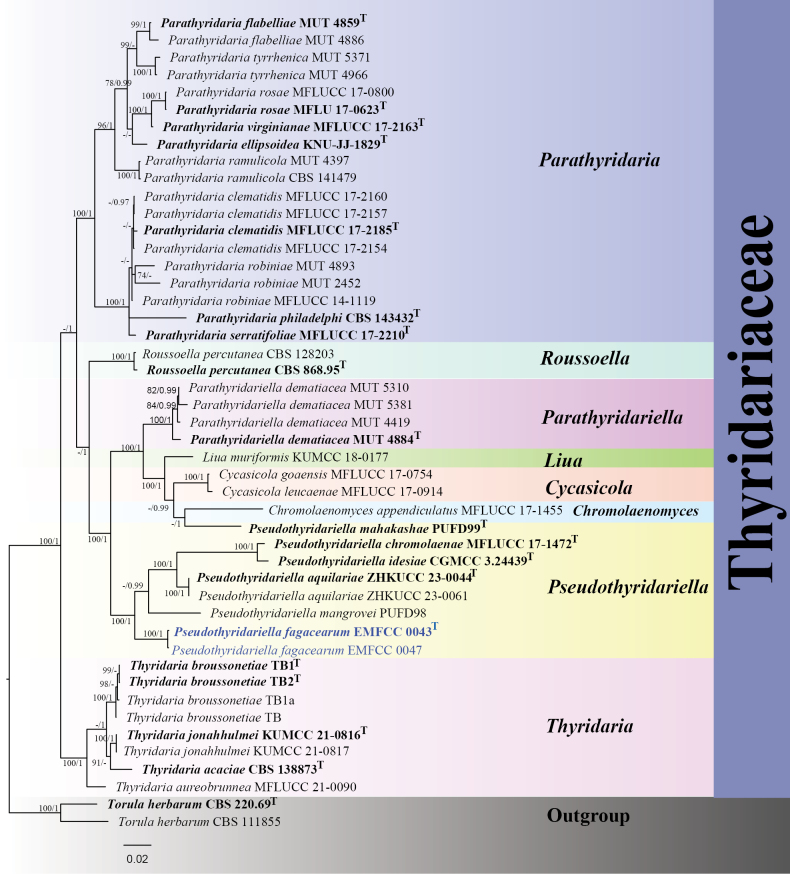
The Bayesian 50% majority-rule consensus phylogram based on a concatenated ITS, LSU, SSU, *rpb*2, and *tef*1-α of Thyridariaceae. The tree was rooted with *Torula herbarum* (CBS 111855) and *T. herbarum* (CBS 595.96). Bootstrap support values for maximum likelihood greater than 70% (ML left) and Bayesian posterior probabilities ≥ 0.90 (BPP right) are shown at the nodes. The new isolates are indicated in blue. The type-derived strains are indicated in bold.

The single-locus and multi-locus phylogenetic analyses (ITS, LSU, SSU, *rpb*2, and *tef*1-α) were implemented to elucidate the phylogenetic position of the isolates EMFCC 0043 and EMFCC 0047. In the single-locus phylogenetic trees of ITS and *rpb*2 genes, they exhibited similar topologies (Suppl. material 2: figs S1, S2). The delineation of genera within the family Thyridariaceae is clear in each of these trees, and the species within the genus *Pseudothyridariella* can be distinctly differentiated. In contrast, the phylogenetic relationships inferred from the LSU, SSU, and *tef*1-α genes demonstrated limited resolution. The tree topologies generated from ML and BI analyses showed nearly complete congruence (Suppl. material 2: figs S3, S4). The results revealed that EMFCC 0043 and EMFCC 0047 represent a previously undescribed species within *Pseudothyridariella*, which we formally proposed as *Pseudothyridariella fagacearum*. This taxon formed a distinct and monophyletic lineage together with *P. aquilariae* (ZHKUCC 23‐0044), *P. chromolaenae* (MFLUCC 17-1472), *P. idesiae* (CGMCC 3.24439), and *P. mangrovei* (PUFD98) with high statistical support (100% ML, 1.00 BPP) (Fig. 1).

#### ﻿Phylogenetic analyses of *Phaeoseptum*

**Figure 2.**
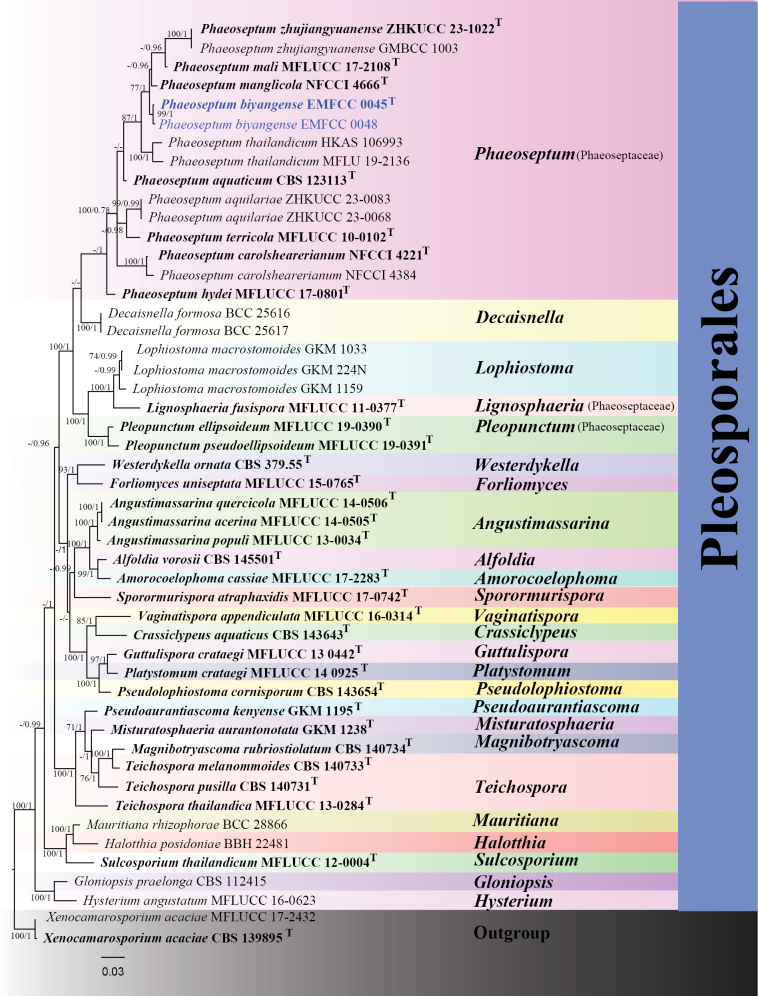
The Bayesian 50% majority-rule consensus phylogram based on a concatenated ITS, LSU, SSU, and *tef*1-α of Phaeoseptaceae. The tree was rooted with *Xenocamarosporium acaciae* (MFLUCC 17-2432) and *X. acaciae* (CBS 139895). Bootstrap support values for maximum likelihood greater than 70% (ML left) and Bayesian posterior probabilities ≥ 0.90 (BPP right) are shown at the nodes. The new isolates are indicated in blue. The type-derived strains are indicated in bold.

In the individual ITS tree, our isolates EMFCC 0045 and EMFCC 0048 were grouped with *P. zhujiangyuanense* (ZHKUCC 23-1022, GMBCC1003), *P. manglicola* (NFCCI 4666), and *P. mali* (MFLUCC 17-2108) (Suppl. material 2: fig. S5). Consequently, the ITS gene region may not provide adequate phylogenetic signal to resolve species boundaries among these closely related *Phaeoseptum* taxa. In the individual LSU tree, the species of *Phaeoseptum* are scattered and do not form an independent group within Phaeoseptaceae. The SSU gene provides limited support for the generic-level delineation within Phaeoseptaceae (Suppl. material 2: fig. S6). Nevertheless, the phylogenetic tree based on the *tef*1-α gene regions clearly distinguished the isolates EMFCC 0045 and EMFCC 0048 from their sister species with 61% ML support (Suppl. material 2: fig. S7). Multi-locus phylogenetic analyses using concatenated sequences of ITS, LSU, SSU, and *tef*1-α genes showed that the isolates EMFCC 0045 and EMFCC 0048 clustered with *P. zhujiangyuanense* (GMBCC1003, ZHKUCC 23-1022), *P. mali* (MFLUCC 17-2108), and *P. manglicola* (NFCCI 4666) with strong support (77% ML/1.00 BPP) (Fig. 2). These results underscore the efficacy of the *tef*1-α marker in resolving evolutionary relationships and species boundaries within the genus.

#### ﻿Phylogenetic analyses of *Byssosphaeria*

**Figure 3.**
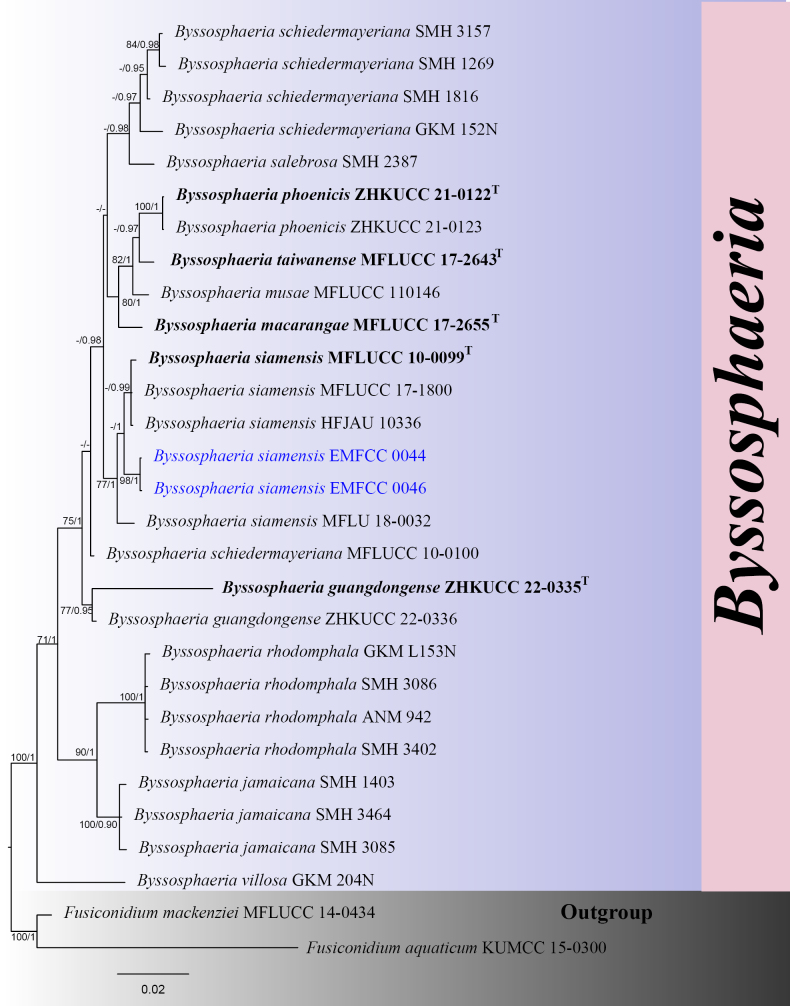
The Bayesian 50% majority-rule consensus phylogram based on a concatenated ITS, LSU, SSU, and *tef*1-α of *Byssosphaeria*. The tree was rooted with *Fusiconidium aquaticum* (MFLUCC 14-0434) and *F. mackenziei* (KUMCC 15-0300). Bootstrap support values for maximum likelihood greater than 70% (ML left) and Bayesian posterior probabilities ≥ 0.90 (BPP right) are shown at the nodes. The new isolates are indicated in blue. The type-derived strains are indicated in bold.

In the single-locus phylogeny, ITS and *tef*1-α were reliable genetic markers for discriminating *Byssosphaeria* species (Suppl. material 2: figs S8, S9). Multilocus phylogenetic analyses showed that the isolates EMFCC 0044 and EMFCC 0046 clustered in a well-supported clade (77% ML /1.00 BPP) comprising four strains of *B. siamensis* (MFLUCC 17-1800, MFLUCC 10-0099, MFLU 18-0032, and HFJAU10336) (Fig. 3).

### ﻿Taxonomy

#### ﻿ Pseudothyridariella fagacearum

Taxon classificationFungiPleosporalesThyridariaceae

W.X. Su, R. Xu & X. Li sp. nov.

97577987-EBBF-59B7-8081-BBD578B017EC

856426

Fig. 4

##### Etymology.

Refers to the host family, Fagaceae.

##### Description.

***Saprobic*** on dead stems of Fagaceae. ***Sexual morph*: *Ascomata*** 110–254 × 145–320 µm (x̄ = 171 × 213 µm, n = 5), solitary or scattered, immersed, bulge on the branches, visible as black spots, coriaceous, globose to subglobose, brown, ostiolate. ***Peridium*** 27.8–32.3 µm wide, thick, multi-layered, composed of 4–6 layers of brown to pale brown cells of ***textura angularis***. ***Hamathecium*** comprising 1.9–3.27 µm wide, dense, filiform, branched, transversely septate, hyaline, cellular pseudoparaphyses, embedded in a gelatinous matrix. ***Asci*** 88–138 × 24–36 µm (x̄ = 113 × 30 µm, n = 15), 8-spored, cylindric, bitunicate, fissitunicate, straight or slightly curved, with a short pedicellate. ***Ascospores*** 47–66 × 13–23 µm (x̄ = 58 × 19 µm, n = 30), hyaline, 1–2-seriate, partially overlapping, straight or slightly curved, ellipsoid to broadly fusiform, smooth-walled, tapering towards both ends, 8-septate, constricted at the central septum, the septum breaks from the middle and the contents overflow when mature, with a hyaline gelatinous sheath. ***Asexual morph***: Undetermined.

##### Culture characteristics.

Ascospores germinate on PDA within 24 h at 25 °C in the dark. Colonies on PDA, reaching 46–52 mm after 2 weeks at 25 °C. Culture from above, circular, asperulate, dense, mycelium slightly raised, tangerine in the center, pale orange radiating outward, margin was undulate to petaloid, zonate, with concentric rings; reverse dark orange, pale yellow at margin. Yellow pigmentation diffused into the media.

##### Material examined.

China, • Jiangxi Province, Anfu, from the branches of Fagaceae, 14 June 2023, Wenxin Su, AF01 (HMJAU 70074, holotype); ex-type living culture, EMFCC 0043; ex-isotype living culture, EMFCC 0047.

##### GenBank accession number.

EMFCC 0043: ITS = PQ557514, SSU = PQ557516, LSU = PQ530968, *rpb*2 = PQ736597, *tef*1-α = PQ683825. EMFCC 0047: ITS = PV463747, SSU = PV490596, LSU = PV490599, and *rpb*2 = PV670039.

##### Notes.

In a BLASTn search, the ITS sequence of *Pseudothyridariella fagacearum* (EMFCC 0043) was 96.21% similar to *P. chromolaenae* (MFLUCC 17-1472). The LSU and SSU regions showed 98.73% and 98.97% similarity with those of *P. aquilariae* (ZHKUCC 23–0044). The *rpb*2 sequence of *P. fagacearum* showed 86.87% similarity with *Pseudothyridariella* sp. JM-2024a, while the *tef*1-α sequence displayed 96.49% similarity with *Pseudothyridariella* sp. (RL-2023a). In the phylogenetic analyses, our isolates of *P. fagacearum* (EMFCC 0043 and EMFCC 0047) formed a well-separated lineage distinct in *Pseudothyridariella* with high statistical support (83% ML/0.99 BPP).

The genus *Pseudothyridariella* currently comprises two asexual species, *P. aquilariae* and *P. idesiae* (Li et al. 2023; Tian et al. 2024), and two sexual morph species, *P. chromolaenae* and *P. mahakoshae* (Devadatha et al. 2018; Mapook et al. 2020). *Pseudothyridariella fagacearum* can be distinguished from *P. chromolaenae* and *P. mahakoshae* by larger asci (70–220 × 10–20 µm vs. 88–138 × 24–36 µm vs. 80–135 × 14–22 µm) and larger ascospores (47–66 × 13–23 µm vs. 23–28 × 9–12.5 µm vs. 17–27 × 5–12 µm). Additionally, the ascospores of *P. fagacearum* are hyaline, 8-septate, and lacking a longitudinal septum. In contrast, the ascospores of *P. chromolaenae* are brown, olivaceous brown to dark brown at maturity, with 5–8 transverse septa and 1 longitudinal septum (Mapook et al. 2020), while those of *P. mahakoshae* are hyaline, with 3–6 transverse septa and 1 longitudinal septum (Devadatha et al. 2018). Therefore, based on both phylogenetic and morphological evidence, *P. fagacearum* was described herein as a new species.

**Figure 4.**
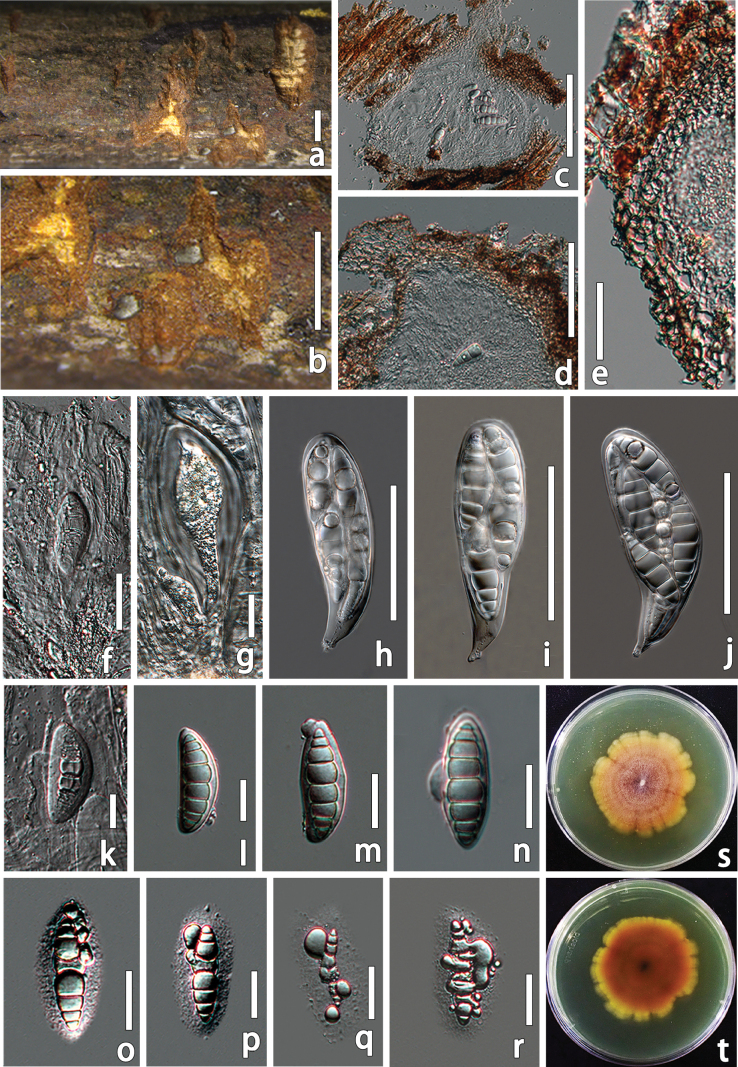
*Pseudothyridariella fagacearum* (HMJAU 70074, holotype). a, b. Ascomata on host surface; c. Vertical section of partial ascoma; d. Ostioles; e. Peridium; f–j. Asci; k–r. Ascospores; s. Cultural characteristics of the PDA in front; t. Cultural characteristics of the PDA in back. Scale bars: 1 mm (a, b); 100 µm (c, d); 20 µm (e, g–k); 50 µm (f).

#### ﻿ Phaeoseptum biyangense

Taxon classificationFungiPleosporalesPhaeoseptaceae

W.X. Su, R. Xu & X. Li sp. nov.

620C5D2B-BEFE-52B2-AB13-A80596AEE091

856427

Fig. 5

##### Etymology.

Refers to the type locality, Biyang County.

##### Description.

***Saprobic*** on dead branches of Fagaceae. ***Sexual morph*: *Ascomata*** 415–445 × 467–606 μm (x̄ = 432 × 525 µm, n = 5), solitary to gregarious, depressed, immersed, globose to subglobose, irregular, convex, without ostiole. ***Peridium*** 18–25 μm (x̄ = 23.5 μm, n = 10) wide, multi-layered, comprising 5–9 layers of cells of ***textura angularis***, thick-walled, dark brown to brown cells of outer layers, and hyaline cells of inner layers. ***Hamathecium*** comprising 1–2.2 μm (x̄ = 1.4 µm, n = 20) wide, dense, numerous, branched, cellular pseudoparaphyses, extending above the asci, embedded in a gelatinous matrix. ***Asci*** 152–170 × 25–32 μm (x̄ = 164 × 27 μm, n = 20), 8-spored, bitunicate, fissitunicate, cylindrical to clavate, apically rounded, with an ocular chamber, long pedicellate. ***Ascospores*** 34–47 × 9–12 μm (x̄ = 39 × 11 μm, n = 30), overlapping, 1–2-seriate, oblong to broadly fusiform, slightly curved, initially hyaline, becoming pale to yellowish brown, smooth-walled, muriform, with 9–18-transverselly septa, 0–4 longitudinal septa in each row, slightly constricted at septum, cell above median septum slightly enlarged, tapering towards both ends, broadly rounded or truncate at apex and base, guttulate, without sheath or appendages. Asexual morph: undetermined.

##### Culture characteristics.

Single ascospores germinate on PDA within 24 h. Colonies growing on PDA at 25 °C, dark, reaching 37 mm diam after four weeks. Cultures from above, circular, dense, gray, curled, floccose, umbonate, dentate, and iron gray at the margins; reverse was dark gray, with white, sparse hyphae radiating outward.

**Figure 5.**
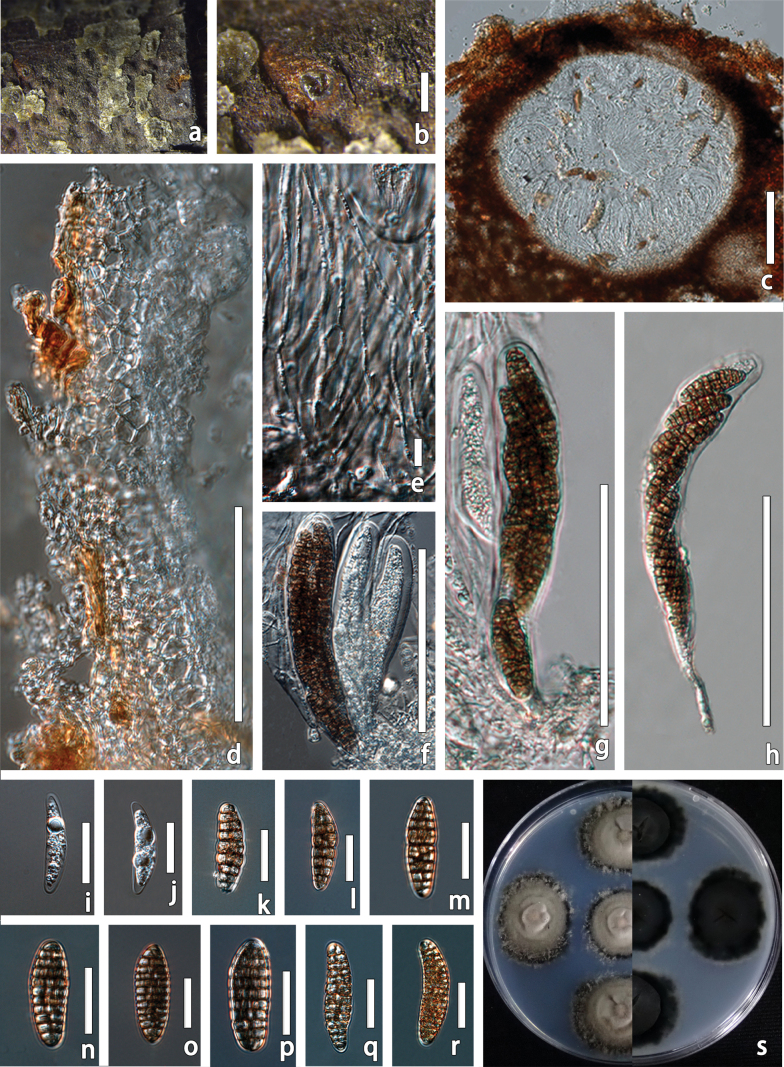
*Phaeoseptum biyangense* (HMJAU 70076, holotype). a, b. Ascomata on host surface; c. Vertical section through ascoma; d. Peridium; e. Pseudoparaphyses; f–h. Asci; i–r. Ascospores; s. Front and back view of culture in PDA. Scale bars: 500 µm (b); 100 µm (c, f–h); 50 µm (d); 5 µm (e); 20 µm (i–r).

##### Material examined.

China, • Henan Province, Biyang, from the branches of Fagaceae, 12 June 2023, Wenxin Su, HN02 (HMJAU 70076, holotype); ex-type living culture, EMFCC 0045; ex-isotype living culture, EMFCC 0048.

##### GenBank accession number.

EMFCC 0045: ITS = PQ686747, SSU = PQ686299, LSU = PQ686300, *tef*1-α = PQ724421. EMFCC 0048: ITS = PV463748, SSU = PV490598, LSU = PV490600, and *tef*1-α = PV670038.

##### Notes.

Morphological comparisons revealed that the ascomata of *Phaeoseptum biyangense* are considerably larger than those of related species, *P. zhujiangyuanense*, *P. mali*, and *P. manglicola* (415–445 × 467–606 μm *vs.* 215–470 × 150–320 μm *vs.* 320–375 × 320–360 μm *vs.* 210–420 × 170–374 μm) (Phukhamsakda 2019; Dayarathne 2020; Zhang et al. 2024). The ascospores of *P. biyangense* possess more septa (9–18 vs. 7–13 *vs.* 11–14 vs. 9–13) and wider dimensions compared to *P. zhujiangyuanense*, *P. mali*, and *P. manglicola* (34–47 × 9–12 *vs.* 35–42 × 9–15 *vs.* 27–38 × 8–13 *vs.* 27–36 × 7.5–13). Notably, *P. biyangense* lacks distinct ostioles (Zhang et al. 2017, 2024; Phukhamsakda 2019; Dayarathne 2020; Jayawardena et al. 2022). In the multi-locus phylogeny, *P. biyangense* showed close affinity to *P. manglicola* (NFCCI 4666). The ITS, LSU, SSU, and *tef*1-α base pair differences between two species (excluding gaps) are 3/413 bp (0.61%), 3/790 bp (0.38%), 198/1034 bp (11.91%), and 3/757 bp (0.40%), respectively. Thus, we describe *P. biyangense* as a novel species.

#### ﻿ Byssosphaeria siamensis

Taxon classificationFungiPleosporalesMelanommataceae

Boonmee, Q. Tian & K.D. Hyde, Fungal Diversity 74: 283 (2015)

DEEE0D61-36BA-5F17-BF96-FFFEFC7BB1A9

Index Fungorum: IF551430

Facesoffungi Number: FoF01026

Fig. 6

##### Description.

***Saprobic*** on decaying wood of Fagaceae. ***Sexual morph*: *Ascomata*** 586–690 × 425–630 μm (x̄ = 638 × 527 µm, n = 5), scattered, solitary, mostly superficial, unilocular, globose to subglobose, covered with dense dark brown setae (2–6 μm wide), dark brown to black, coriaceous, ostiolate. ***Ostiole*** central, wide, with a pore-like opening visible on the host, orange to yellow. ***Peridium*** 54–69 μm wide, multilayered, heavily pigmented, comprising 13–16 layers, hyaline to dark brown cells of ***textura angularis*** to ***textura prismatica***. ***Hamathecium*** comprising 1.5–3.3 μm wide, dense, septate, filiform, branching pseudoparaphyses, embedded in a gelatinous matrix. ***Asci*** 115–146 × 9–12 μm (x̄ = 127 × 10 μm, n = 20), 8-spored, bitunicate, fissitunicate, cylindrical to clavate, apically rounded, with a well-developed ocular chamber, long pedicellate (46–53 μm long). ***Ascospores*** 31.6–37.6 × 6–7.6 μm (x̄ = 34 × 7 μm, n = 20), biseriate, overlapping, oblong fusiform, hyaline to pale brown, tapering towards both ends, smooth-walled, slightly curved, 1-septate, constricted at the septa, without mucilaginous sheath. Asexual morph: Undetermined.

##### Culture characteristics.

Single ascospores germinate on MEA within 24 h. Colonies on MEA, fast growing, full plate within one week at 25 °C, medium dense, convex, velvety with white aerial mycelium, entire, reverse dark orange, pale yellow at margin. Orange pigmentation diffused into the media.

##### Material examined.

China, • Jiangxi Province, Anfu, from the branches of Fagaceae, 14 June 2023, Wenxin Su, AF06 (HMJAU 70074); living culture, EMFCC 0044 and EMFCC 0046.

##### GenBank accession number.

EMFCC 0044: ITS = PQ686747, LSU = PQ686300, SSU = PQ686299. EMFCC 0046: ITS = PV463746, LSU = PV490601, SSU = PV490597, and *tef*1-α = PV670037.

##### Notes.

*Byssosphaeria siamensis* was originally discovered from decaying wood of an unidentified host in Thailand (Tian et al. 2018). Subsequently, this species has been documented on *Pandanus*, *Citrus trifoliata*, and submerged decaying wood (Hyde et al. 2018; Tian et al. 2018; Tibpromma et al. 2018; Tennakoon et al. 2024). In the present study, our specimen was isolated from withered branches of Fagaceae plants in Jiangxi, China. Morphological examination revealed that the ascomata of these strains are consistent with previous descriptions, although subtle variations were observed in ascospore morphology (Hyde et al. 2018; Tian et al. 2018; Tibpromma et al. 2018; Tennakoon et al. 2024) (Suppl. material 1: table S2). In the phylogenetic analysis, the isolate (EMFCC 0044 and EMFCC 0046) formed a well-supported clade with *B. siamensis* (MFLUCC 17-1800, MFLU 17-1004, MFLU 18-0032, HFJAU10336). Therefore, we identified our collection as *B. siamensis*, and this is a new host record from Fagaceae in China.

**Figure 6.**
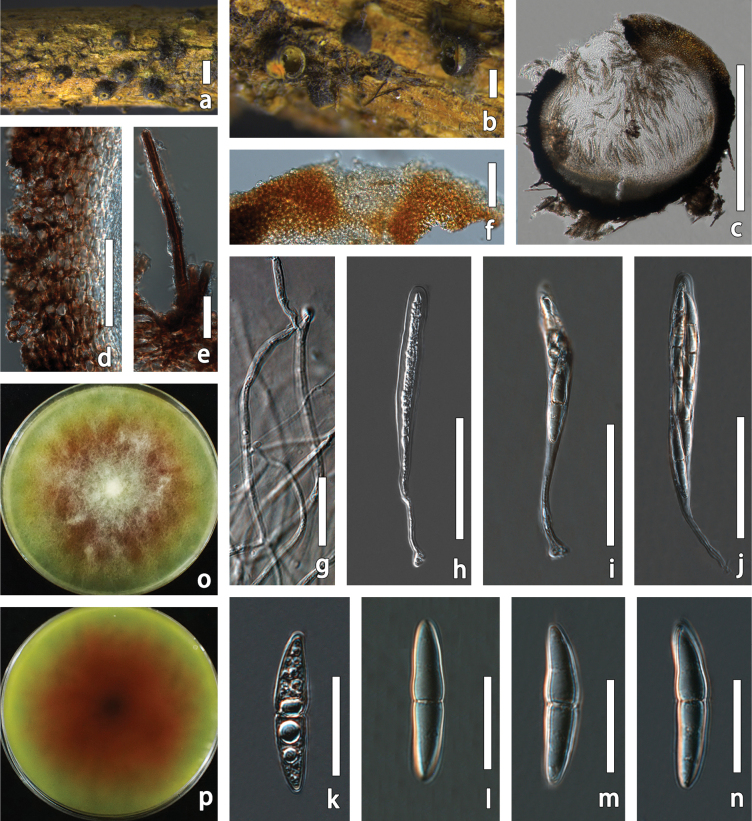
*Byssosphaeria siamensis* (HMJAU 70075). a, b. Ascomata on host surface; c. Vertical section through partial ascoma; d. Peridium; e. Setae on the ascomata; f. Ostiole; g. Pseudoparaphyses; h–j. Asci; k–n. Ascospores; o. Cultural characteristics of the PDA in front; p. Cultural characteristics of the PDA in back. Scale bars: 1 mm (a); 500 µm (b, c); 50 µm (d, f, h–j); 20 µm (e, g, k–n).

## ﻿Discussion

*Pseudothyridariella* was established by Mapook et al. (2020) as a new genus in Thyridariaceae, which differs from *Thyridariella* in having ascospores with constriction at the central septum (Devadatha et al. 2018; Mapook et al. 2020). The genus was typified by *Pseudothyridariella chromolaenae* Mapook & K.D. Hyde (Mapook et al. 2020). Based on the Species Fungorum (www.speciesfungorum.org), there are four epithets (*P. aquilariae* T.Y. Du, Tibpromma & Karun., *P. chromolaenae*, *P. idesiae* W.L. Li & Jian K. Liu, and *P. mahakoshae* (Devadatha, V.V. Sarma, D.N. Wanas., K.D. Hyde & E.B.G. Jones) Mapook and K.D. Hyde) listed under this genus. In this study, we introduce *P. fagacearum* as a novel species, isolated from dead stems of Fagaceae in Jiangxi Province, China. *Pseudothyridariella fagacearum* forms a phylogenetically distinct clade and is morphologically distinguishable by the absence of longitudinal septa and the presence of larger asci and ascospores compared to its close relatives. The genus *Pseudothyridariella* exhibits a broad geographical distribution and a diversity of plant hosts. Specifically, *P. aquilariae* was documented on dead branches of *Aquilaria sinensis* (Thymelaeaceae) in Guangdong Province, China (Tian et al. 2024). *Pseudothyridariella chromolaenae* was discovered from dead twigs of *Chromolaena odorata* (Asteraceae) in Thailand (Mapook et al. 2020), while *P. mahakoshae* was found on dead twigs of *Avicennia marina* (Acanthaceae) in India (Devadatha et al. 2018). Additionally, *P. idesiae* was reported on dead twigs of *Idesia polycarpa* (Salicaceae) in Sichuan Province, China (Li et al. 2023). These species have been recorded in tropical and subtropical regions, suggesting a potential preference for warm and humid environments among *Pseudothyridariella* species.

Zhang et al. (2012) proposed *Phaeoseptum* as a monotypic genus in Halotthiaceae and designated *P. aquaticum* Ying Zhang, J. Fourn. & K.D. Hyde as the type species (Zhang et al. 2012). Hyde et al. (2018) transferred *Phaeoseptum* to Phaeoseptaceae based on the evidence of taxonomy and phylogeny. There are nine species accepted in this genus (www.speciesfungorum.org). The genus is characterized by semi-immersed to immersed ascomata, cellular pseudoparaphyses, muriform and brown ascospores (Zhang et al. 2017; Hyde et al. 2018; Phukhamsakda 2019; Dayarathne 2020; Jayawardena et al. 2022; Zhang et al. 2024). Based on single-gene analyses, both the generic circumscription of *Phaeoseptum* and the classification of its constituent species remain unresolved (Hyde et al. 2018). To address these taxonomic challenges, the application of a multi-locus phylogenetic analysis approach is anticipated to provide a more robust resolution.

*Byssosphaeria*, a member of the family Melanommataceae (Pleosporales, Ascomycota), was erected by Cooke and Plowright (1879) with *B. keithii* as the type species. *Byssosphaeria* is characterized by superficial, separate, or gregarious ascomata with bright yellow, orange, or red flat apices around the ostiole, with a minute rounded ostiole and a coelomycetous anamorph (Barr 1990; Hawksworth et al. 1995; Tennakoon et al. 2018). *Byssosphaeria* species are predominantly saprobes inhabiting diverse plant substrates (*Alnus* sp., *Bambusa* sp., *Erythrina indica*, *Litsea* sp., *Musa* sp., *Quercus* sp., and *Salix* sp.) (Barr 1984; Sivanesan and Hsieh 1989; Chen and Hsieh 2004; Wang et al. 2004; Medel 2007; Li and Zhuang 2008; Liu et al. 2015) and have a cosmopolitan distribution (e.g., Austria, Brazil, China, the Czech Republic, France, Jamaica, Mexico, Poland, Sweden, Thailand, and the USA) (Kirk 1983; Kirk et al. 2001; Zhang et al. 2012; Tennakoon et al. 2018; Hyde et al. 2013; Wanasinghe et al. 2018; Jayawardena et al. 2022; Kularathnage et al. 2022; Xiong et al. 2023). To date, five strains of *B. siamensis* (MFLUCC 17-1800, MFLUCC 10-0099, MFLU 18-0032, HFJAU 10336, and HMJAU 70074) have been reported in Thailand and China, exhibiting a diverse host range, including *Citrus trifoliata* (Rutaceae) and *Pandanus* sp. (Pandanaceae) (Zhang et al. 2017; Hyde et al. 2018; Tian et al. 2018; Tibpromma et al. 2018; Jayawardena et al. 2022; Tennakoon et al. 2024). The present study represents the first report of *B. siamensis* associated with Fagaceae. Subtle differences in ascospore morphology were documented among host-associated strains (Suppl. material 1: table S2), suggesting potential host-mediated influences on fungal morphogenesis. The newly identified strain contributes additional molecular data to *Byssosphaeria*. Nevertheless, taxonomic confusion remains unresolved (Kularathnage et al. 2022), underscoring the need for additional morphological and molecular data to clarify species boundaries.

## Supplementary Material

XML Treatment for Pseudothyridariella fagacearum

XML Treatment for Phaeoseptum biyangense

XML Treatment for Byssosphaeria siamensis

## ﻿Additional information

### Use of AI

No use of AI was reported.

### Author ORCIDs

Wen-Xin Su https://orcid.org/0000-0002-5470-5853

Ranagul Tieliwadi https://orcid.org/0009-0007-1322-3296

Wen-Ying Su https://orcid.org/0000-0002-4687-2854

Xiao Li https://orcid.org/0000-0002-1230-6467

Rong Xu https://orcid.org/0000-0002-7744-6321

### Data availability

All sequences generated in this study were submitted to GenBank, and all of the data from this study are available in the main text or supplementary information.

## Supplementary materials

Supplementary material 1 Supplementary tables Data type docx Explanation note **table S1.** Names, strain numbers, and corresponding GenBank accession numbers of taxa were used in this study. **table S2.** Different strain morphological characteristics of *Byssosphaeria siamensis*. https://binary.pensoft.net/file/1410060This dataset is made available under the Open Database License (http://opendatacommons.org/licenses/odbl/1.0/). The Open Database License (ODbL) is a license agreement intended to allow users to freely share, modify, and use this Dataset while maintaining this same freedom for others, provided that the original source and author(s) are credited.Wen-Xin Su, Ranagul Tieliwadi, Wen-Ying Su, Xiao Li, Rong Xu

Supplementary material 2 Supplementary figures Data type docx Explanation note **figure S1.** The best-scoring RAxML tree based on a concatenated ITS dataset of Thyridariaceae. **figure S2.** The best-scoring RAxML tree based on a concatenated rpb2 dataset of Thyridariaceae. **figure S3.** The best-scoring RAxML tree based on a concatenated ITS, LSU, SSU, rpb2 and tef1-α dataset of Thyridariaceae. **figure S4.** Phylogram generated from Bayesian inference analysis based on combined ITS, LSU, SSU, rpb2 and tef1-α dataset of Thyridariaceae. **figure S5.** The best-scoring RAxML tree based on a concatenated ITS dataset of Phaeoseptaceae. **figure S6.** The best-scoring RAxML tree based on a concatenated SSU dataset of Phaeoseptaceae. **figure S7.** Phylogram generated from maximum parsimony analysis based on combined *tef*1-α dataset of Phaeoseptaceae. **figure S8.** The best-scoring RAxML tree based on a concatenated ITS dataset of *Byssosphaeria*. **figure S9.** Phylogram generated from maximum parsimony analysis based on combined tef1-α dataset of *Byssosphaeria*. https://binary.pensoft.net/file/1410061This dataset is made available under the Open Database License (http://opendatacommons.org/licenses/odbl/1.0/). The Open Database License (ODbL) is a license agreement intended to allow users to freely share, modify, and use this Dataset while maintaining this same freedom for others, provided that the original source and author(s) are credited.Wen-Xin Su, Ranagul Tieliwadi, Wen-Ying Su, Xiao Li, Rong Xu
