# Supplementary material for: ﻿Additions to woody litter fungi of Byssosphaeria, Phaeoseptum and Pseudothyridariella (Pleosporales, Ascomycota) from China
Source: MycoKeys. 2025 Sep 5;122:35–57. doi: 10.3897/mycokeys.122.161224 (PMC12432522; doi:10.3897/mycokeys.122.161224)
Supplement: Supplementary material 1 — Supplementary tables [file mycokeys-122-035-s001.docx]

**Additions to Woody Litter Fungi of *Phaeoseptum*, *Pseudothyridariella* and *Byssosphaeria* (Pleosporales, Ascomycota) from China**

WenXin Su^1^, Ranagul·Tieliwadi^1^, WenYing Su^1,3^, Xiao Li^1*^ and Rong Xu^1,2^*

**1 Joint International Research Laboratory of Modern Agricultural Technology, Ministry of Education, Jilin Agricultural University, Changchun, 130118, China; suwenxin1220@163.com(W.X.S); [m15526846682@163.com](mailto:m15526846682@163.com)(R.T.);lxmogu@163.com(X.L.)**

**2 School of Food Science and Engineering, Yangzhou University, Yangzhou, 225127, China; xurong@jlau.edu.cn(R.X)**

**3 Lianyungang Academy of Agricultural Sciences, Lianyungang, 222006, China; 18004425758@163.com(W.Y.S)**

**Correspondence: lxmogu@163.com(X.L.); xurong@jlau.edu.cn(R.X.)**

**Table 1:** Names, strain numbers, and corresponding GenBank accession numbers of taxa were used in this study.

**Table 2:** Different strain morphological characteristics of *Byssosphaeria siamensis*.

| Table 1. Names, strain numbers, and corresponding GenBank accession numbers of taxa were used in this study. | | | | | | | | | | | | | |
| --- | --- | --- | --- | --- | --- | --- | --- | --- | --- | --- | --- | --- | --- |
| **Taxon** | | **Strain Number** | | **GenBank Accession Numbers** | | | | | | | | | |
|  |  |  |  | **ITS** | | | **LSU** | | **SSU** | | tef1-α | | ***rpb*2** |
| *Parathyridaria clematidis* | | MFLUCC 17-2157 | | MT310644 | | | MT214600 | | MT226711 | | MT394656 | | MT394711 |
| *P. clematidis* | | MFLUCC 17-2160 | | MT310643 | | | MT214599 | | MT226710 | | MT394655 | | MT394710 |
| *P. clematidis* | | MFLUCC 17-2185 | | MT310642 | | | MT214598 | | MT226709 | | MT394654 | | MT394709 |
| *P. clematidis* | | MFLUCC 17-2154 | | MT310645 | | | MT214601 | | MT226712 | | MT394657 | | MT394712 |
| *P. robiniae* | | MUT 4893 | | KM355998 | | | MN556328 | | KM355993 | | MN605904 | | MN605924 |
| *P. robiniae* | | MFLUCC 14-1119 | | KY511142 | | | KY511141 | | _ | | KY549682 | | _ |
| *P. philadelphi* | | CBS 143432 | | MH107905 | | | MH107951 | | _ | | MH108023 | | _ |
| *P. serratifoliae* | | MFLUCC 17-2210 | | MT310646 | | | MT214602 | | MT226713 | | MT394658 | | MT394713 |
| *P. tyrrhenica* | | MUT 4966 | | KR014366 | | | KP671740 | | _ | | MN605911 | | MN605931 |
| *P. tyrrhenica* | | MUT 5371 | | KU314951 | | | MN556329 | | KU314952 | | MN605912 | | MN605932 |
| *P. robiniae* | | MUT 2452 | | MG813183 | | | MG816491 | | _ | | MN605903 | | MN605923 |
| *P. flabelliae* | | MUT 4859 | | _ | | | KP671716 | | KT587315 | | MN605909 | | MN605929 |
| *P. flabelliae* | | MUT 4886 | | _ | | | KP671720 | | KT587317 | | MN605910 | | MN605930 |
| *P. rosae* | | MFLU 17-0623 | | NR_157530 | | | NG_059873 | | _ | | _ | | _ |
| *P. rosae* | | MFLUCC 17-0800 | | MG828940 | | | MG829049 | | _ | | _ | | _ |
| *P. virginia e* | | MFLUCC 17-2163 | | MT310647 | | | MT214603 | | MT226714 | | MT394659 | | MT394714 |
| *P. ellipsoidea* | | KNU JJ 1829 | | LC552950 | | | LC552952 | | _ | | _ | | _ |
| *P. ramulicola* | | CBS 141479 | | NR_147657 | | | _ | | NG_061254 | | _ | | _ |
| *P. ramulicola* | | MUT 4397 | | KC339235 | | | KF636775 | | MN556311 | | MN605913 | | MN605933 |
| *P. percutanea* | | CBS 128203 | | KF322117 | | | KF366448 | | KF366450 | | KF407988 | | KF366453 |
| *P. percutanea* | | CBS 868 95 | | KF322118 | | | KF366449 | | KF366451 | | KF407987 | | KF366452 |
| *Cycasicola goaensis* | | MFLUCC 17-0754 | | MG828885 | | | MG829001 | | MG829112 | | MG829198 | | _ |
| *C. leucaee* | | MFLUCC 17-0914 | | NR 163322 | | | NG 070071 | | NG 065771 | | MK360046 | | MK434900 |
| *Liua muriformis* | | KUMCC 18-0177 | | MK433599 | | | MK433598 | | MK433595 | | MK426798 | | MK426799 |
| *Chromolaenomyces appendiculatus* | | MFLUCC 17-1455 | | MT214346 | | | MT214440 | | MT214394 | | MT235770 | | MT235806 |
| *Parathyridariella dematiacea* | | MUT 5381 | | KU314959 | | | MN556331 | | KU314960 | | MN605908 | | MN605928 |
| *Pa. dematiacea* | | MUT5310 | | KU255057 | | | MN556330 | | MN556314 | | MN605907 | | MN605927 |
| *Pa. dematiacea* | | MUT 4419 | | KC339245 | | | KF636786 | | MN556313 | | MN605905 | | MN605925 |
| *Pa. dematiacea* | | MUT4884 | | NR_169701 | | | _ | | NG_070286 | | _ | | _ |
| *Thyridariella mangrovei* | | PUFD98 | | MG020434 | | | MG020437 | | MG020440 | | MG020443 | | MG020445 |
| *Pseudothyridariella idesiae* | | CGMCC 3.24439 | | OR253148 | | | OR253307 | | OR253216 | | OR251154 | | OR253762 |
| *Ps.aquilariae* | | ZHKUCC 23‐0044 | | OR825376 | | | PP809728 | | PP809708 | | PP812259 | | PP812241 |
| *Ps.aquilariae* | | ZHKUCC 23‐0611 | | OR825377 | | | PP809729 | | PP809709 | | PP812260 | | PP812242 |
| *Ps. chromolae e* | | MFLUCC 17-1472 | | MT214347 | | | MT214441 | | MT214395 | | MT235771 | | MT235807 |
| *Ps. mahakoshae* | | PUFD99 | | MG020435 | | | MG020438 | | MG020441 | | MG023140 | | MG020446 |
| *Ps. fagacearum* | | EMFCC 0043 | | PQ557514 | | | PQ530968 | | PQ557516 | | PQ736597 | | PQ683825 |
| *Ps. fagacearum* | | EMFCC 0047 | | PV463747 | | | PV490599 | | PV490596 | | PV670039 | | _ |
| *Thyridaria broussonetiae* | | TB2 | | KX650570 | | | _ | | _ | | KX650540 | | KX650587 |
| *T. broussonetiae* | | TB1 | | KX650568 | | | _ | | KX650515 | | KX650539 | | KX650586 |
| *T. broussonetiae* | | TB1a | | KX650569 | | | _ | | _ | | _ | | _ |
| *T. broussonetiae* | | TB | | KX650567 | | | _ | | _ | | KX650538 | | KX650585 |
| *T. johhulmei* | | KUMCC 21-0817 | | ON007042 | | | ON007038 | | ON007047 | | ON009132 | | ON009136 |
| *T. johhulmei* | | KUMCc 21-0816 | | ON007041 | | | ON007037 | | ON007046 | | ON009131 | | ON009135 |
| *T. acaciae* | | CBS 138873 | | KP004469 | | | KP004497 | | _ | | _ | | _ |
| *T. aureobrunnea* | | MFLUCC 21-0090 | | NR_182937 | | | NG_088276 | | _ | | _ | | _ |
| *Torula herbarum* | | CBS 220.69 | |  | | |  | | KF443389 | | KF443401 | | KF443393 |
| *To. herbarum* | | CBS 111855 | | KF443409 | | | _ | | KF443391 | | KF443403 | | KF443396 |
| **Phaeoseptaceae** | | | | | | | | | | | | | |
| **Taxon** | **Strain numbers** | | | | **ITS** | | | **LSU** | | **SSU** | | **tef1-α** | |
| *Alfoldia vorosii* | CBS 145501 | | | | [JN859336](http://www.ncbi.nlm.nih.gov/nuccore/JN859336) | | | [MK589354](http://www.ncbi.nlm.nih.gov/nuccore/MK589354) | | [MK589346](http://www.ncbi.nlm.nih.gov/nuccore/MK589346) | | [MK599320](http://www.ncbi.nlm.nih.gov/nuccore/MK599320) | |
| *Amorocoelophoma cassiae* | MFLUCC 17-2283 | | | | [NR_163330](http://www.ncbi.nlm.nih.gov/nuccore/NR_163330) | | | [NG_066307](http://www.ncbi.nlm.nih.gov/nuccore/NG_066307) | | [NG_065775](http://www.ncbi.nlm.nih.gov/nuccore/NG_065775) | | [MK360041](http://www.ncbi.nlm.nih.gov/nuccore/MK360041) | |
| *Angustimassari aceri* | MFLUCC 14-0505 | | | | [NR_138406](http://www.ncbi.nlm.nih.gov/nuccore/NR_138406) | | | [KP888637](http://www.ncbi.nlm.nih.gov/nuccore/KP888637) | | [NG_063573](http://www.ncbi.nlm.nih.gov/nuccore/NG_063573) | | [KR075168](http://www.ncbi.nlm.nih.gov/nuccore/KR075168) | |
| *A.populi* | MFLUCC 13-0034 | | | | [KP899137](http://www.ncbi.nlm.nih.gov/nuccore/KP899137) | | | [KP888642](http://www.ncbi.nlm.nih.gov/nuccore/KP888642) | | [NG_061204](http://www.ncbi.nlm.nih.gov/nuccore/NG_061204) | | [KR075164](http://www.ncbi.nlm.nih.gov/nuccore/KR075164) | |
| *A. quercicola* | MFLUCC 14-0506 | | | | [KP899133](http://www.ncbi.nlm.nih.gov/nuccore/KP899133) | | | [KP888638](http://www.ncbi.nlm.nih.gov/nuccore/KP888638) | | [NG_063574](http://www.ncbi.nlm.nih.gov/nuccore/NG_063574) | | [KR075169](http://www.ncbi.nlm.nih.gov/nuccore/KR075169) | |
| *Crassiclypeus aquaticus* | CBS 143643 | | | | [LC312501](http://www.ncbi.nlm.nih.gov/nuccore/LC312501) | | | [LC312530](http://www.ncbi.nlm.nih.gov/nuccore/LC312530) | | [LC312472](http://www.ncbi.nlm.nih.gov/nuccore/LC312472) | | [LC312559](http://www.ncbi.nlm.nih.gov/nuccore/LC312559) | |
| *Decaisnella formosa* | BCC 25616 | | | | _ | | | [GQ925846](http://www.ncbi.nlm.nih.gov/nuccore/GQ925846) | | [GQ925833](http://www.ncbi.nlm.nih.gov/nuccore/GQ925833) | | [GU479851](http://www.ncbi.nlm.nih.gov/nuccore/GU479851) | |
| *D. formosa* | BCC 25617 | | | | _ | | | [GQ925847](http://www.ncbi.nlm.nih.gov/nuccore/GQ925847) | | [GQ925834](http://www.ncbi.nlm.nih.gov/nuccore/GQ925834) | | [GU479850](http://www.ncbi.nlm.nih.gov/nuccore/GU479850) | |
| *Forliomyces uniseptata* | MFLUCC 15-0765 | | | | [NR_154006](http://www.ncbi.nlm.nih.gov/nuccore/NR_154006) | | | [NG_059659](http://www.ncbi.nlm.nih.gov/nuccore/NG_059659) | | [NG_061234](http://www.ncbi.nlm.nih.gov/nuccore/NG_061234) | | [KU727897](http://www.ncbi.nlm.nih.gov/nuccore/KU727897) | |
| *Gloniopsispraelonga* | CBS 112415 | | | | _ | | | [FJ161173](http://www.ncbi.nlm.nih.gov/nuccore/FJ161173) | | [FJ161134](http://www.ncbi.nlm.nih.gov/nuccore/FJ161134) | | [FJ161090](http://www.ncbi.nlm.nih.gov/nuccore/FJ161090) | |
| *Guttulispora crataegi* | MFLUCC 13-0442 | | | | [KP899134](http://www.ncbi.nlm.nih.gov/nuccore/KP899134) | | | [KP888639](http://www.ncbi.nlm.nih.gov/nuccore/KP888639) | | [KP899125](http://www.ncbi.nlm.nih.gov/nuccore/KP899125) | | [KR075161](http://www.ncbi.nlm.nih.gov/nuccore/KR075161) | |
| *Halotthiaposidoniae* | BBH 22481 | | | | _ | | | [GU479786](http://www.ncbi.nlm.nih.gov/nuccore/GU479786) | | [GU479752](http://www.ncbi.nlm.nih.gov/nuccore/GU479752) | | _ | |
| *Hysterium angustatum* | MFLUCC 16-0623 | | | | _ | | | [FJ161180](http://www.ncbi.nlm.nih.gov/nuccore/FJ161180) | | [GU397359](http://www.ncbi.nlm.nih.gov/nuccore/GU397359) | | [FJ161096](http://www.ncbi.nlm.nih.gov/nuccore/FJ161096) | |
| *Lignosphaeria fusispora* | MFLUCC 11-0377 | | | | [NR_164233](http://www.ncbi.nlm.nih.gov/nuccore/NR_164233) | | | [KP888646](http://www.ncbi.nlm.nih.gov/nuccore/KP888646) | | _ | | _ | |
| *Mauritia rhizophorae* | BCC 28866 | | | | _ | | | [GU371824](http://www.ncbi.nlm.nih.gov/nuccore/GU371824) | | [GU371832](http://www.ncbi.nlm.nih.gov/nuccore/GU371832) | | [GU371817](http://www.ncbi.nlm.nih.gov/nuccore/GU371817) | |
| *Misturatosphaeria aurantiacinotata* | GKM 1238 | | | | _ | | | [NG_059927](http://www.ncbi.nlm.nih.gov/nuccore/NG_059927) | | _ | | [GU327761](http://www.ncbi.nlm.nih.gov/nuccore/GU327761) | |
| *Phaeoseptum aquaticum* | CBS 123113 | | | | [KY940803](http://www.ncbi.nlm.nih.gov/nuccore/KY940803) | | | [JN644072](http://www.ncbi.nlm.nih.gov/nuccore/JN644072) | | _ | | _ | |
| *Ph. aquilariae* | ZHKUCC 23‐0068 | | | | OR825380 | | | PP809732 | | PP809712 | | PP812261 | |
| *Ph. aquilariae* | ZHKUCC 23‐0083 | | | | OR825381 | | | PP809733 | | _ | | PP812262 | |
| *Ph. carolshearerianum* | NFCCI 4221 | | | | [MK307810](http://www.ncbi.nlm.nih.gov/nuccore/MK307810) | | | [MK307813](http://www.ncbi.nlm.nih.gov/nuccore/MK307813) | | [MK307816](http://www.ncbi.nlm.nih.gov/nuccore/MK307816) | | [MK309874](http://www.ncbi.nlm.nih.gov/nuccore/MK309874) | |
| *Ph. carolshearerianum* | NFCCI 4384 | | | | [MK307812](http://www.ncbi.nlm.nih.gov/nuccore/MK307812) | | | [MK307815](http://www.ncbi.nlm.nih.gov/nuccore/MK307815) | | [MK307818](http://www.ncbi.nlm.nih.gov/nuccore/MK307818) | | [MK309876](http://www.ncbi.nlm.nih.gov/nuccore/MK309876) | |
| *Ph. hydei* | MFLUCC 17-0801 | | | | [MT240622](http://www.ncbi.nlm.nih.gov/nuccore/MT240622) | | | [MT240623](http://www.ncbi.nlm.nih.gov/nuccore/MT240623) | | [MT240624](http://www.ncbi.nlm.nih.gov/nuccore/MT240624) | | [MT241506](http://www.ncbi.nlm.nih.gov/nuccore/MT241506) | |
| *Ph. mali* | MFLUCC 17-2108 | | | | [MK659580](http://www.ncbi.nlm.nih.gov/nuccore/MK659580) | | | [MK625197](http://www.ncbi.nlm.nih.gov/nuccore/MK625197) | | _ | | [MK647990](http://www.ncbi.nlm.nih.gov/nuccore/MK647990) | |
| *Ph. manglicola* | NFCCI 4666 | | | | [MK307811](http://www.ncbi.nlm.nih.gov/nuccore/MK307811) | | | [MK307814](http://www.ncbi.nlm.nih.gov/nuccore/MK307814) | | [MK307817](http://www.ncbi.nlm.nih.gov/nuccore/MK307817) | | [MK309875](http://www.ncbi.nlm.nih.gov/nuccore/MK309875) | |
| *Ph. terricola* | MFLUCC 10-0102 | | | | [MH105778](http://www.ncbi.nlm.nih.gov/nuccore/MH105778) | | | [MH105779](http://www.ncbi.nlm.nih.gov/nuccore/MH105779) | | [MH105780](http://www.ncbi.nlm.nih.gov/nuccore/MH105780) | | [MH105781](http://www.ncbi.nlm.nih.gov/nuccore/MH105781) | |
| *Ph. thailandicum* | MFLU 19-2136 | | | | [OM293749](http://www.ncbi.nlm.nih.gov/nuccore/OM293749) | | | [OR211590](http://www.ncbi.nlm.nih.gov/nuccore/OR211590) | | [OM293755](http://www.ncbi.nlm.nih.gov/nuccore/OM293755) | | [OM305059](http://www.ncbi.nlm.nih.gov/nuccore/OM305059) | |
| *Ph. thailandicum* | HKAS 106993 | | | | [OM293750](http://www.ncbi.nlm.nih.gov/nuccore/OM293750) | | | [OM293745](http://www.ncbi.nlm.nih.gov/nuccore/OM293745) | | [OM293756](http://www.ncbi.nlm.nih.gov/nuccore/OM293756) | | [OM305060](http://www.ncbi.nlm.nih.gov/nuccore/OM305060) | |
| *Ph. zhujiangyuanense* | ZHKUCC 23-1022 | | | | [PP060500](http://www.ncbi.nlm.nih.gov/nuccore/PP060500) | | | [PP060514](http://www.ncbi.nlm.nih.gov/nuccore/PP060514) | | [PP060506](http://www.ncbi.nlm.nih.gov/nuccore/PP060506) | | [PP035541](http://www.ncbi.nlm.nih.gov/nuccore/PP035541) | |
| *Ph. zhujiangyuanense* | GMBCC 1003 | | | | [PP067152](http://www.ncbi.nlm.nih.gov/nuccore/PP067152) | | | [PP067157](http://www.ncbi.nlm.nih.gov/nuccore/PP067157) | | [PP066044](http://www.ncbi.nlm.nih.gov/nuccore/PP066044) | | [PP068813](http://www.ncbi.nlm.nih.gov/nuccore/PP068813) | |
| *Ph. biyangense* | EMFCC 0045 | | | | PQ557515 | | | PQ530969 | | PQ557517 | | PQ724421 | |
| *Ph. biyangense* | EMFCC 0048 | | | | PV463748 | | | PV490600 | | PV490598 | | PV670038 | |
| *Platystomum crataegi* | MFLUCC 14-0925 | | | | [KT026117](http://www.ncbi.nlm.nih.gov/nuccore/KT026117) | | | [KT026109](http://www.ncbi.nlm.nih.gov/nuccore/KT026109) | | [KT026113](http://www.ncbi.nlm.nih.gov/nuccore/KT026113) | | [KT026121](http://www.ncbi.nlm.nih.gov/nuccore/KT026121) | |
| *Pl. ellipsoideum* | MFLUCC 19-0390 | | | | [MK804512](http://www.ncbi.nlm.nih.gov/nuccore/MK804512) | | | [MK804517](http://www.ncbi.nlm.nih.gov/nuccore/MK804517) | | [MK804514](http://www.ncbi.nlm.nih.gov/nuccore/MK804514) | | [MK828510](http://www.ncbi.nlm.nih.gov/nuccore/MK828510) | |
| *Pl. pseudoellipsoideum* | MFLUCC 19-0391 | | | | [MK804513](http://www.ncbi.nlm.nih.gov/nuccore/MK804513) | | | [MK804518](http://www.ncbi.nlm.nih.gov/nuccore/MK804518) | | _ | | [MK828511](http://www.ncbi.nlm.nih.gov/nuccore/MK828511) | |
| *Pseudoaurantiascoma kenyense* | GKM 1195 | | | | _ | | | [NG_059928](http://www.ncbi.nlm.nih.gov/nuccore/NG_059928) | | _ | | [GU327767](http://www.ncbi.nlm.nih.gov/nuccore/GU327767) | |
| *Ps. cornisporum* | CBS 143654 | | | | [LC312515](http://www.ncbi.nlm.nih.gov/nuccore/LC312515) | | | [LC312544](http://www.ncbi.nlm.nih.gov/nuccore/LC312544) | | [LC312486](http://www.ncbi.nlm.nih.gov/nuccore/LC312486) | | [LC312573](http://www.ncbi.nlm.nih.gov/nuccore/LC312573) | |
| *Ramusculicola thailandica* | MFLUCC 13-0284 | | | | [KP899141](http://www.ncbi.nlm.nih.gov/nuccore/KP899141) | | | [KP888647](http://www.ncbi.nlm.nih.gov/nuccore/KP888647) | | [KP899131](http://www.ncbi.nlm.nih.gov/nuccore/KP899131) | | [KR075167](http://www.ncbi.nlm.nih.gov/nuccore/KR075167) | |
| *Sporormurispora atraphaxidis* | MFLUCC 17-0742 | | | | [NR_157546](http://www.ncbi.nlm.nih.gov/nuccore/NR_157546) | | | [NG_059880](http://www.ncbi.nlm.nih.gov/nuccore/NG_059880) | | [NG_061296](http://www.ncbi.nlm.nih.gov/nuccore/NG_061296) | | _ | |
| *Sulcosporium thailandicum* | MFLUCC 12-0004 | | | | [MG520958](http://www.ncbi.nlm.nih.gov/nuccore/MG520958) | | | [KT426563](http://www.ncbi.nlm.nih.gov/nuccore/KT426563) | | [KT426564](http://www.ncbi.nlm.nih.gov/nuccore/KT426564) | | _ | |
| *Teichospora melanommoides* | CBS 140733 | | | | [NR_154632](http://www.ncbi.nlm.nih.gov/nuccore/NR_154632) | | | [KU601585](http://www.ncbi.nlm.nih.gov/nuccore/KU601585) | | _ | | [KU601610](http://www.ncbi.nlm.nih.gov/nuccore/KU601610) | |
| *T.pusilla* | CBS 140731 | | | | [NR_154633](http://www.ncbi.nlm.nih.gov/nuccore/NR_154633) | | | [KU601586](http://www.ncbi.nlm.nih.gov/nuccore/KU601586) | | _ | | [KU601605](http://www.ncbi.nlm.nih.gov/nuccore/KU601605) | |
| *T. rubriostiolata* | CBS 140734 | | | | [NR_154634](http://www.ncbi.nlm.nih.gov/nuccore/NR_154634) | | | [KU601590](http://www.ncbi.nlm.nih.gov/nuccore/KU601590) | | _ | | [KU601609](http://www.ncbi.nlm.nih.gov/nuccore/KU601609) | |
| *Thyridaria macrostomoides* | GKM 1033 | | | | _ | | | [GU385190](http://www.ncbi.nlm.nih.gov/nuccore/GU385190) | | _ | | [GU327776](http://www.ncbi.nlm.nih.gov/nuccore/GU327776) | |
| *T. macrostomoides* | GKM 1159 | | | | _ | | | [GU385185](http://www.ncbi.nlm.nih.gov/nuccore/GU385185) | | _ | | [GU327778](http://www.ncbi.nlm.nih.gov/nuccore/GU327778) | |
| *T. macrostomoides* | GKM 224N | | | | _ | | | [GU385191](http://www.ncbi.nlm.nih.gov/nuccore/GU385191) | | _ | | [GU327777](http://www.ncbi.nlm.nih.gov/nuccore/GU327777) | |
| *Vagitispora appendiculata* | MFLUCC 16-0314 | | | | [KU743217](http://www.ncbi.nlm.nih.gov/nuccore/KU743217) | | | [KU743218](http://www.ncbi.nlm.nih.gov/nuccore/KU743218) | | [KU743219](http://www.ncbi.nlm.nih.gov/nuccore/KU743219) | | [KU743220](http://www.ncbi.nlm.nih.gov/nuccore/KU743220) | |
| *Westerdykella orta* | CBS 379.55 | | | | [AY943045](http://www.ncbi.nlm.nih.gov/nuccore/AY943045) | | | [GU301880](http://www.ncbi.nlm.nih.gov/nuccore/GU301880) | | [GU296208](http://www.ncbi.nlm.nih.gov/nuccore/GU296208) | | [GU349021](http://www.ncbi.nlm.nih.gov/nuccore/GU349021) | |
| *Xenocamarosporium acaciae* | CBS 139895 | | | | [NR_137982](http://www.ncbi.nlm.nih.gov/nuccore/NR_137982) | | | [NG_058163](http://www.ncbi.nlm.nih.gov/nuccore/NG_058163) | | _ | | _ | |
| *X. acaciae* | MFLUCC 17-2432 | | | | [MK347766](http://www.ncbi.nlm.nih.gov/nuccore/MK347766) | | | [MK347983](http://www.ncbi.nlm.nih.gov/nuccore/MK347983) | | [MK347873](http://www.ncbi.nlm.nih.gov/nuccore/MK347873) | | [MK360093](http://www.ncbi.nlm.nih.gov/nuccore/MK360093) | |
| **Melanommataceae** | | | | | | | | | | | | | |
| **Taxon** | | | **Strain numbers** | | | **ITS** | | **LSU** | | **SSU** | | **tef1-α** | |
| *Byssosphaeria guangdongense* | | | ZHKUCC 22-0336 | | | OQ449321 | | OQ449289 | | OQ449338 | |  | |
| 1. *guangdongense* | | | ZHKUCC 22-0335 | | | OQ449320 | | OQ449288 | | OQ449337 | |  | |
| *B. jamaica* | | | SMH 3464 | | | _ | | GU385153 | | _ | |  | |
| *B. jamaica* | | | SMH 3085 | | | _ | | GU385154 | | _ | | _ | |
| *B. jamaica* | | | SMH 1403 | | | _ | | GU385152 | | _ | | GU327746 | |
| 1. *macarangae* | | | MFLUCC 17-2655 | | | MH389782 | | MH389778 | | MH389780 | | MH389784 | |
| *B. musae* | | | MFLUCC 11-0146 | | | KP744435 | | KP744477 | | KP753947 | | MH581149 | |
| *B. phoenicis* | | | ZHKUCC21-0123 | | | ON180686 | | ON180684 | | ON180692 | | _ | |
| *B. phoenics* | | | ZHKUCC21-0122 | | | ON180685 | | ON180683 | | ON180691 | | _ | |
| *B. rhodomphala* | | | SMH 3086 | | | _ | | GU385155 | | _ | | _ | |
| *B. rhodomphala* | | | ANM 942 | | | _ | | GU385160 | | _ | | _ | |
| *B. rhodomphala* | | | SMH 3402 | | | _ | | GU385170 | | _ | | _ | |
| *B. rhodomphala* | | | GKM L153N | | | _ | | GU385157 | | _ | | GU327747 | |
| *B. salebrosa* | | | SMH 2387 | | | _ | | GU385162 | | _ | | GU327748 | |
| *B. schiedermayeria* | | | MFLUCC 10-0100 | | | _ | | KT289894 | | KT289896 | | _ | |
| *B. schiedermayeria* | | | GKM 152N | | | _ | | GU385168 | | _ | | GU327749 | |
| *B. schiedermayeria* | | | SMH 1816 | | | _ | | GU385159 | | _ | | _ | |
| *B. schiedermayeria* | | | SMH 1269 | | | _ | | GU385158 | | _ | | _ | |
| *B. schiedermayeria* | | | SMH 3157 | | | _ | | GU385163 | | _ | | GU327745 | |
| *B. siamensis* | | | MFLUCC 17-1800 | | | MG543923 | | MG543914 | | MG543917 | | KT962059 | |
| *B. siamensis* | | | MFLUCC 10-0099 | | | _ | | KT289895 | | KT289897 | | _ | |
| *B. siamensis* | | | MFLU 18-0032 | | | MH388334 | | MH376706 | | MH388303 | | MH388370 | |
| *B. siamensis* | | | HFJAU 10336 | | | PP460780 | | PP460773 | | PP460765 | | PP475454 | |
| *B. siamensis* | | | EMFCC 0044 | | | PQ686747 | | PQ686300 | | PQ686299 | | _ | |
| *B. siamensis* | | | EMFCC 0046 | | | PV463746 | | PV490601 | | PV490597 | | PV670037 | |
| *B. taiwanense* | | | MFLUCC 17-2643 | | | MH389783 | | MH389779 | | MH389781 | | MH389785 | |
| *B. willosa* | | | GKM 204N | | | _ | | GU385151 | | _ | | GU327751 | |
| *Fusiconidium aquaticum* | | | KUMCC 15-0300 | | | _ | | KX641894 | | KX641895 | | KX641896 | |
| *F. mackenziei* | | | MFLUCC 14-0434 | | | _ | | KX611112 | | KX611114 | | KX611118 | |

| Table2 Different strain morphological characteristics of *Byssosphaeria siamensis* | | | | | | |
| --- | --- | --- | --- | --- | --- | --- |
| **Culture accession**  **number** | **Local** | **Host** | **Ascomata (**μm**)** | **Asci (**μm**)** | **Ascospores** | **ref** |
| MFLU 10–0099 | THAILAND, Chiang Rai, Muang | decaying wood of unidentified  host | 501–692 × 561–720 | 112–148×10–16 | 40.5–50 × 7–11 μm, biseriate, hyaline to pale yellow, oblong fusiform, 1(−3)-septate, constricted at the septa, slightly curved, surrounded by a gelatinous, fusiform sheath and thickened at both ends, bearing delicate hyaline appendage at ends, wall smooth or verrucose. | (Tian et al. 2015) |
| MFLU 17-1004 | THAILAND, Chiang Rai, Muang, | on submerged  decaying wood in a freshwater stream | 445–475 × 430–555 | 110–150 × 12–14 | 28–33 × 5–7 μm, 1–2-seriate, hyaline to pale brown, ellipsoid to fusiform, 1-septate, constricted at septum, slightly curved, with 1–2 globules, acute at the apex, smooth and thick-walled. | (Hyde et al. 2018) |
| HFJAU10336 | China, Yunnan Province | on a dead stem of *Citrus trifoliata* (Rutaceae) | 250–400 × 300–500 | 110–130 × 11–13 | 30–40 × 6.5–8 μm, overlapping, 1–2-seriate, ellipsoid to fusiform, initially hyaline, pale brown when mature, 1-septate, constricted at the septum, slightly curved, smooth-walled or verrucose. | (Tennakoon et al. 2024) |
| MFLU 18-0032 | THAILAND, Phang Nga Province | on *Pandanu*s sp. (Pandanaceae) | 290–405 9 335–465 | 85–170 9 9– 16 | 30–40 × 5.5–7.5 μm, overlapping 1–2-seriate, fusiform, conical at each end, hyaline to pale brown with age, 1-septate, constricted at the septum, smooth-walled, guttulate, with mucilaginous sheath. | (Tibprommaet al. 2018) |
| EMFCC 0044 | China, Jiangxi Province | on a dead stem of Fagaceae | 586–690 × 425– 630 | 115–146 × 9–12 | 31.6–37.6 × 6–7.6 μm, biseriate, overlapping, hyaline to pale brown, oblong fusiform, tapering towards both ends, smooth-walled, slightly curved, 1–septate, constricted at the septa, lacking mucilaginous sheath. | This study |
