# Supplementary material for: ﻿Additions to woody litter fungi of Byssosphaeria, Phaeoseptum and Pseudothyridariella (Pleosporales, Ascomycota) from China
Source: MycoKeys. 2025 Sep 5;122:35–57. doi: 10.3897/mycokeys.122.161224 (PMC12432522; doi:10.3897/mycokeys.122.161224)
Supplement: Supplementary material 2 — Supplementary figures [file mycokeys-122-035-s002.docx]

**SUPPLEMENTARY MATERIAL FOR**

**Additions to Woody Litter Fungi of *Phaeoseptum*, *Pseudothyridariella* and *Byssosphaeria* (Pleosporales, Ascomycota) from China**

WenXin Su^1^, Ranagul·Tieliwadi^1^, WenYing Su^1,3^, Xiao Li^1*^ and Rong Xu^1,2^*

**1 Joint International Research Laboratory of Modern Agricultural Technology, Ministry of Education, Jilin Agricultural University, Changchun, 130118, China; suwenxin1220@163.com(W.X.S); [m15526846682@163.com](mailto:m15526846682@163.com)(R.T.);lxmogu@163.com(X.L.)**

**2 School of Food Science and Engineering, Yangzhou University, Yangzhou, 225127, China; xurong@jlau.edu.cn(R.X)**

**3 Lianyungang Academy of Agricultural Sciences, Lianyungang, 222006, China; 18004425758@163.com(W.Y.S)**

**Correspondence: lxmogu@163.com(X.L.); xurong@jlau.edu.cn(R.X.)**

**Figure S1:** The best-scoring RAxML tree based on a concatenated ITS dataset of Thyridariaceae.

**Figure S2:** The best-scoring RAxML tree based on a concatenated rpb2 dataset of Thyridariaceae.

**Figure S3:** The best-scoring RAxML tree based on a concatenated ITS, LSU, SSU, rpb2 and tef1-α dataset of Thyridariaceae.

**Figure S4:** Phylogram generated from Bayesian inference analysis based on combined ITS, LSU, SSU, rpb2 and tef1-α dataset of Thyridariaceae.

**Figure S5:** The best-scoring RAxML tree based on a concatenated ITS dataset of Phaeoseptaceae.

**Figure S6:** The best-scoring RAxML tree based on a concatenated SSU dataset of Phaeoseptaceae.

**Figure S7:** Phylogram generated from maximum parsimony analysis based on combined *tef*1-α dataset of Phaeoseptaceae.

**Figure S8:** The best-scoring RAxML tree based on a concatenated ITS dataset of Byssosphaeria.

**Figure S9:** Phylogram generated from maximum parsimony analysis based on combined tef1-α dataset of Byssosphaeria.

**Table S1:** Names, strain numbers, and corresponding GenBank accession numbers of taxa were used in this study.

**Table S2:** Different strain morphological characteristics of Byssosphaeria siamensis.


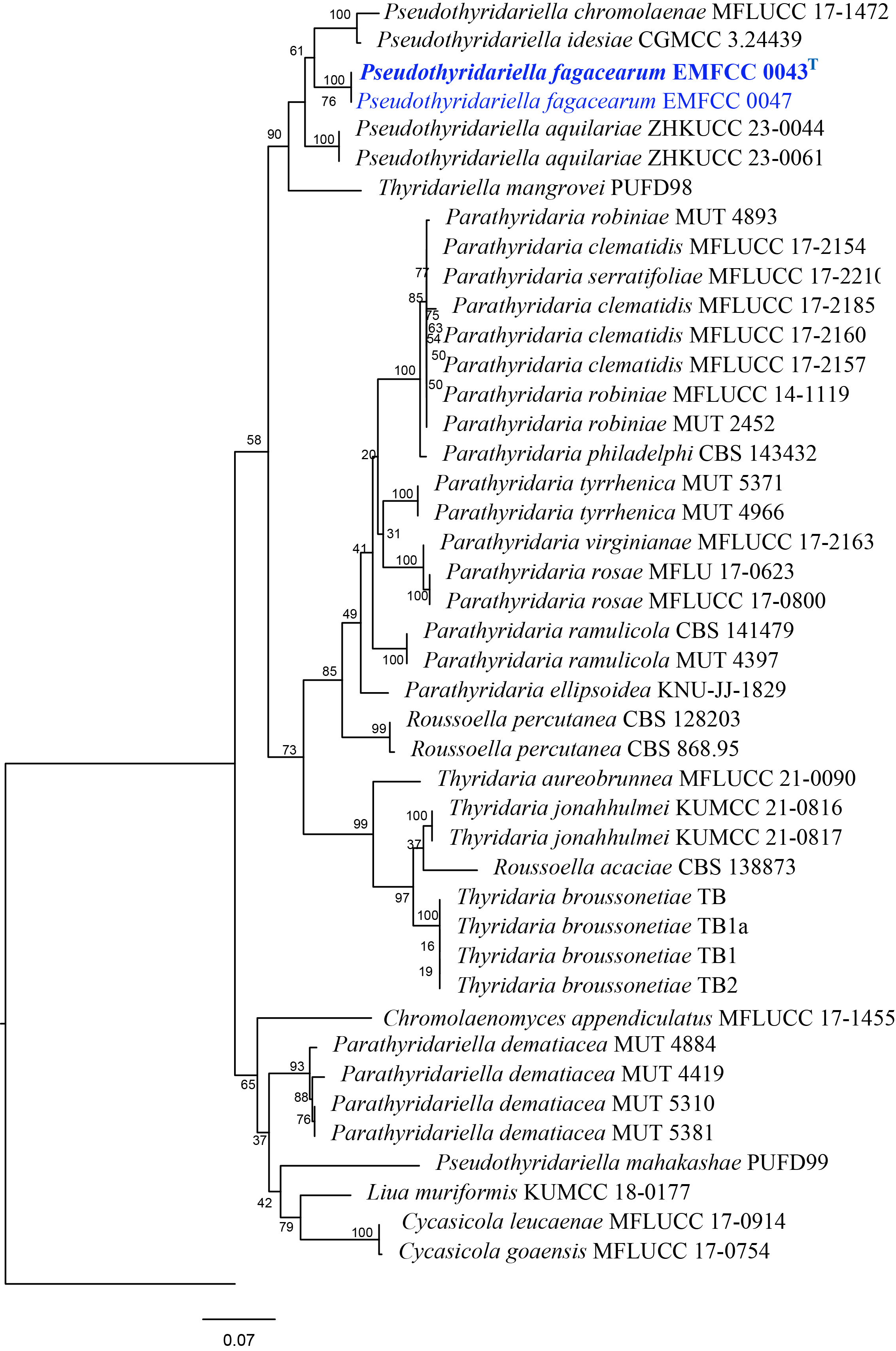


**Figure S1:** The best-scoring RAxML tree based on a concatenated ITS dataset of Thyridariaceae.
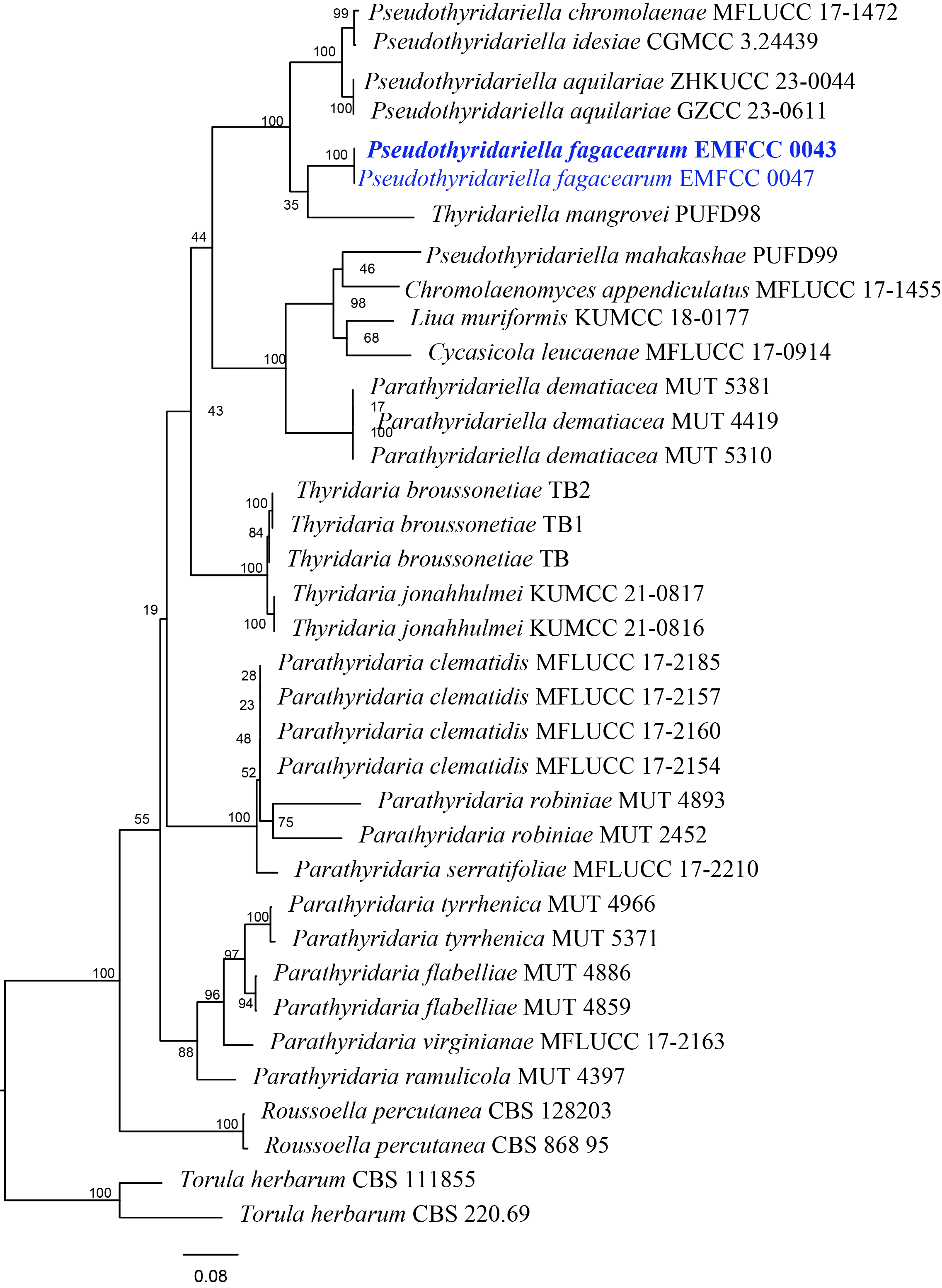


**Figure S2:** The best-scoring RAxML tree based on a concatenated rpb2 dataset of Thyridariaceae.


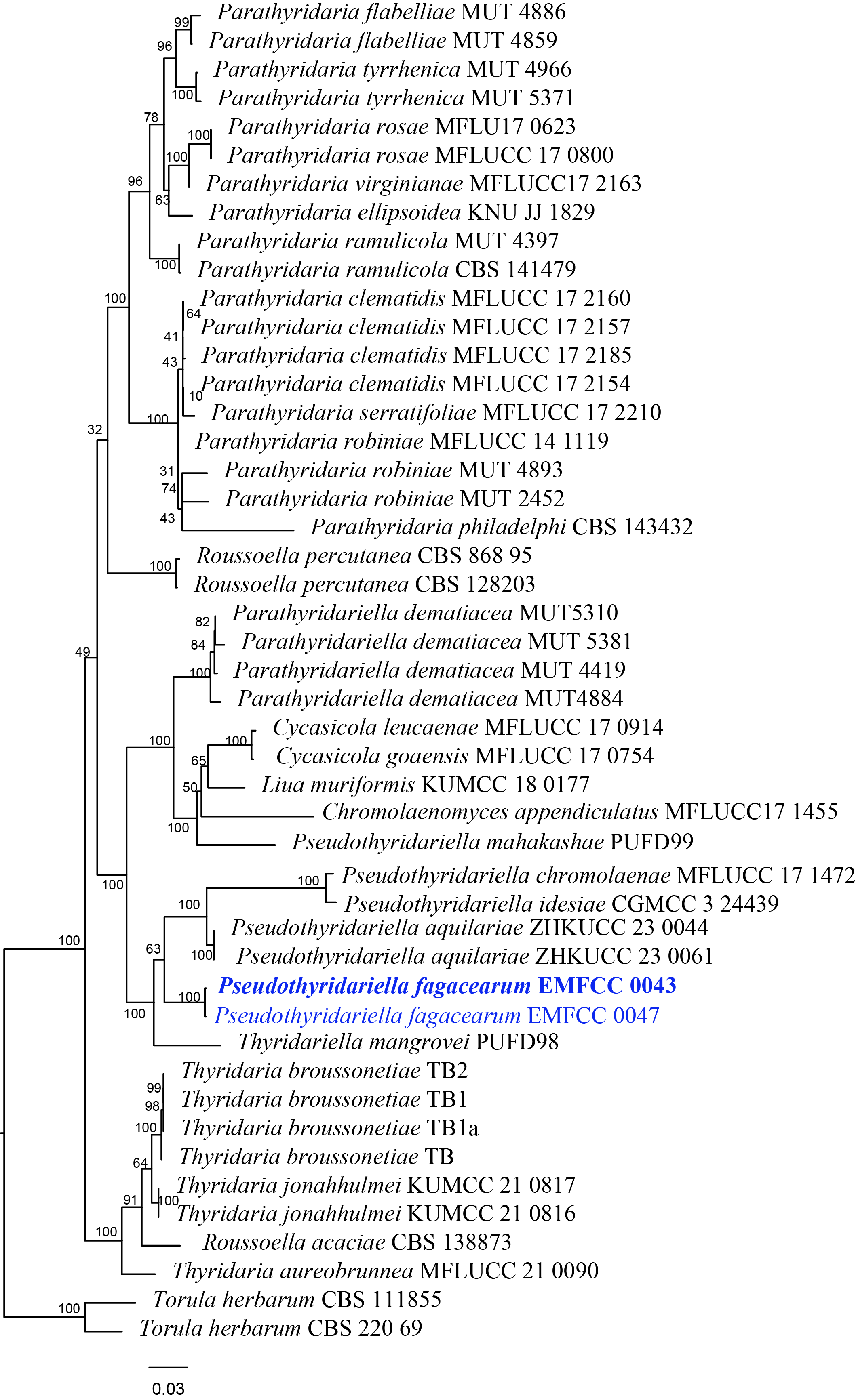


**Figure S3:** The best-scoring RAxML tree based on a concatenated ITS, LSU, SSU, rpb2 and tef1-α dataset of Thyridariaceae.


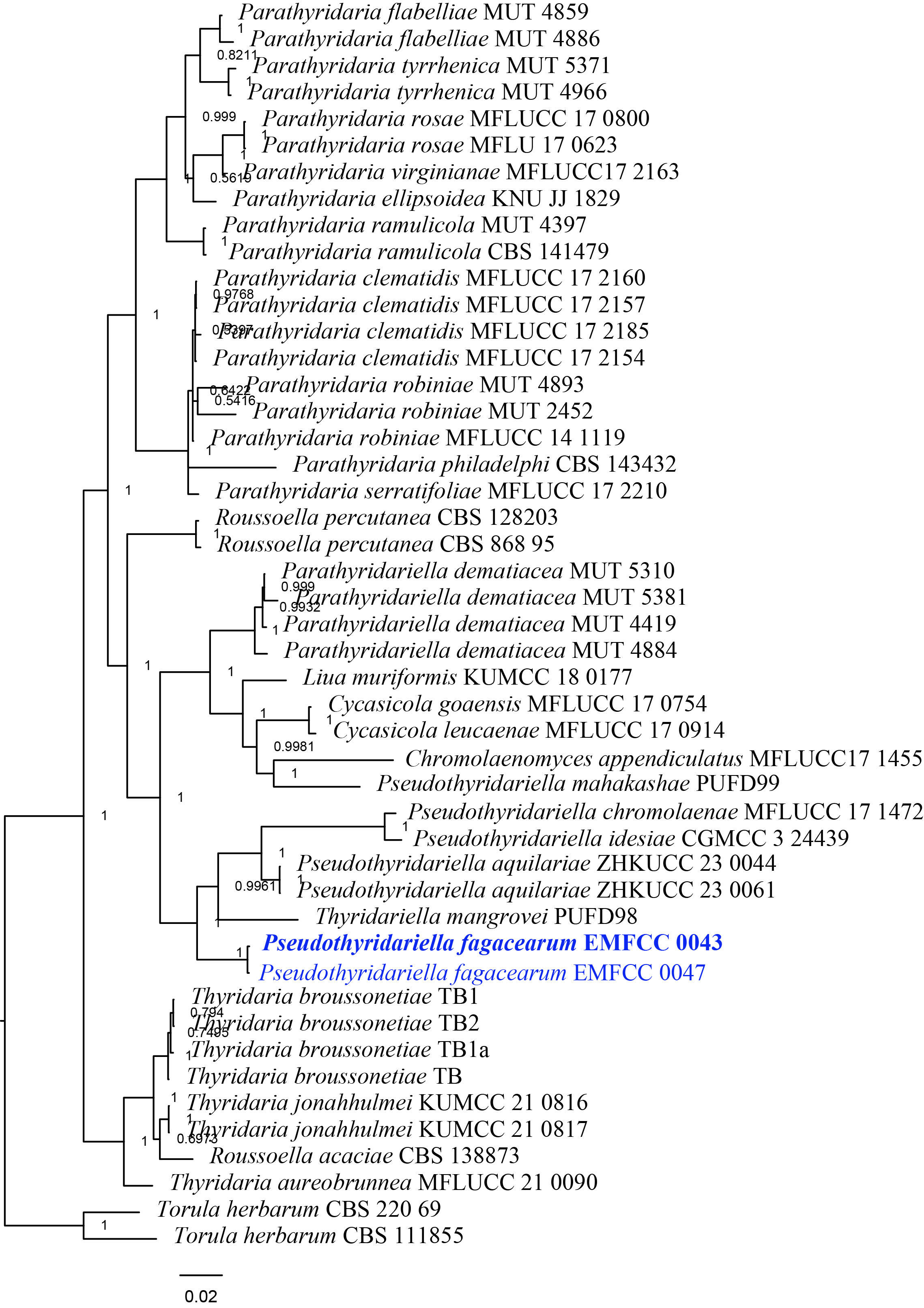


**Figure S4:** Phylogram generated from Bayesian inference analysis based on combined ITS, LSU, SSU, rpb2 and tef1-α dataset of Thyridariaceae.


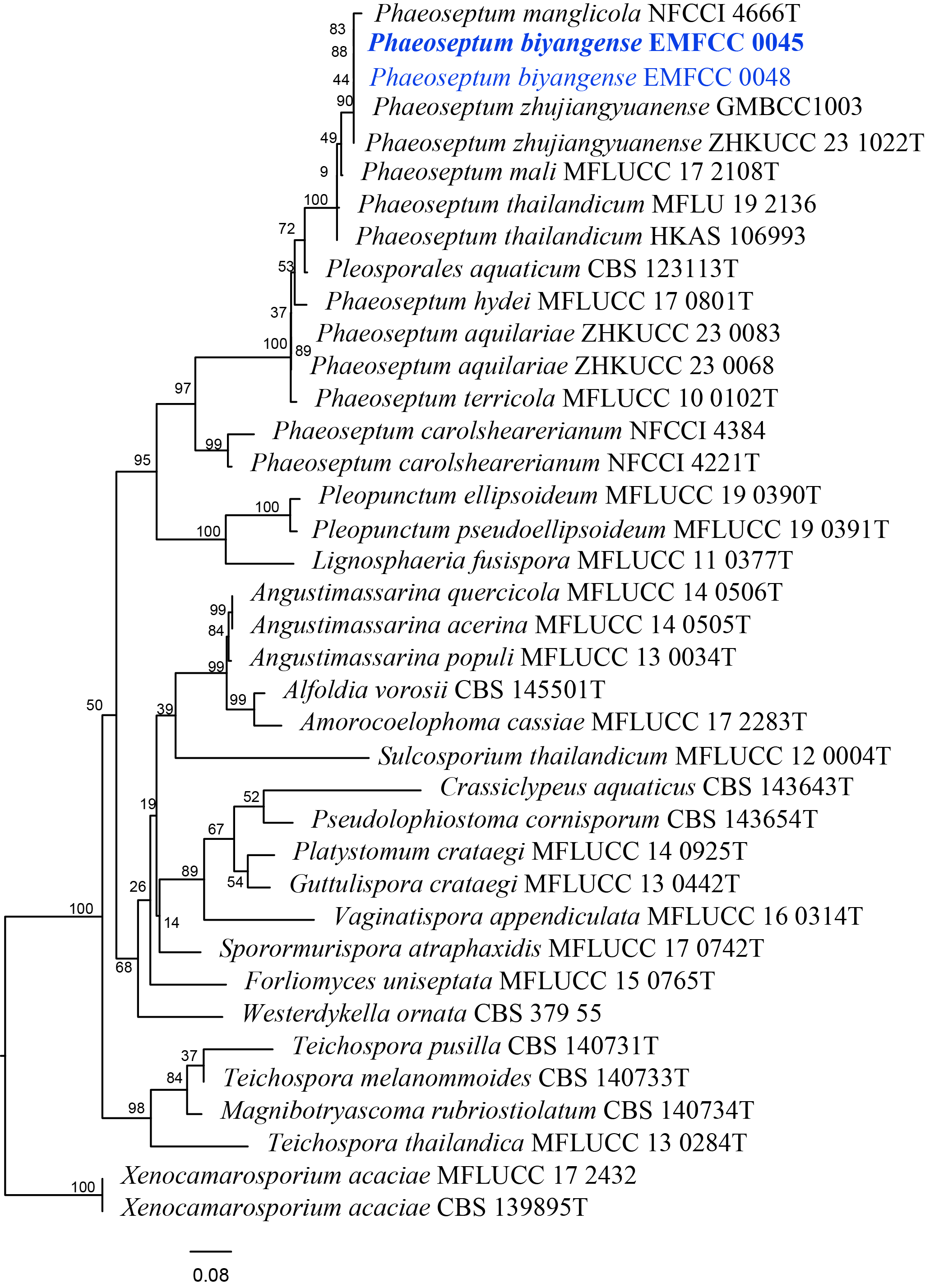


**Figure S5:** The best-scoring RAxML tree based on a concatenated ITS dataset of Phaeoseptaceae.


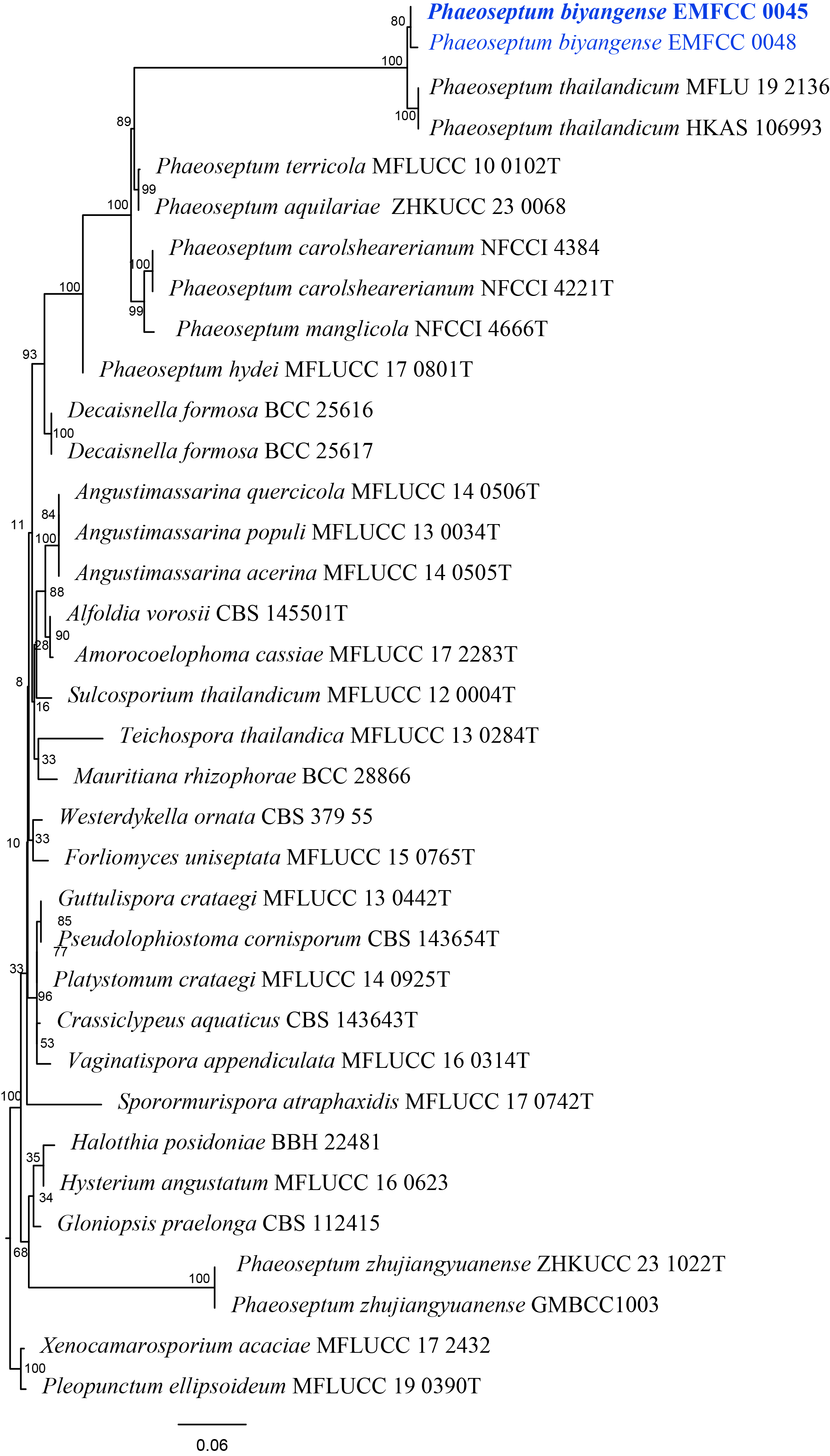


**Figure S6:** The best-scoring RAxML tree based on a concatenated SSU dataset of Phaeoseptaceae.


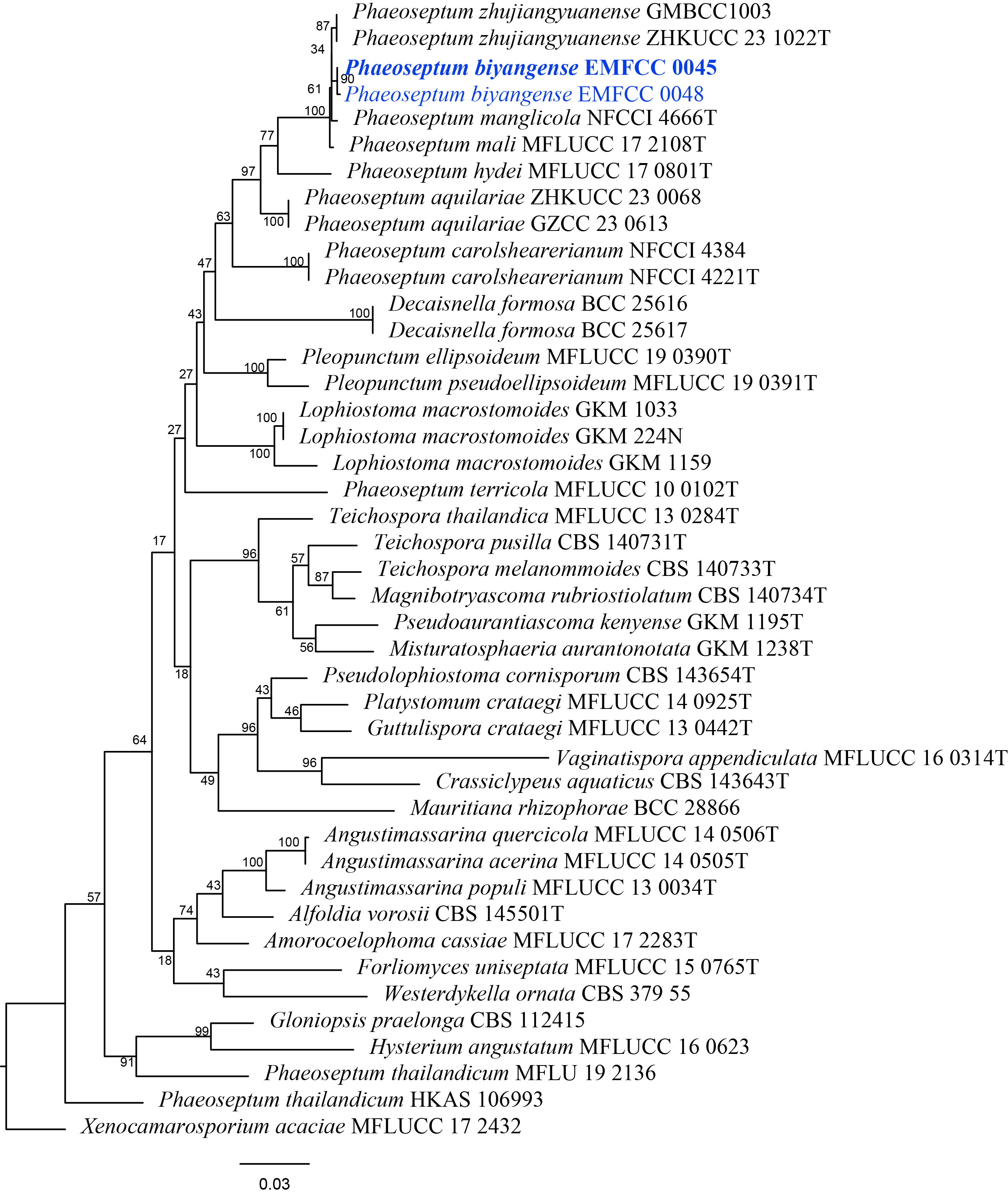


**Figure S7:** Phylogram generated from maximum parsimony analysis based on combined *tef*1-α dataset of Phaeoseptaceae.


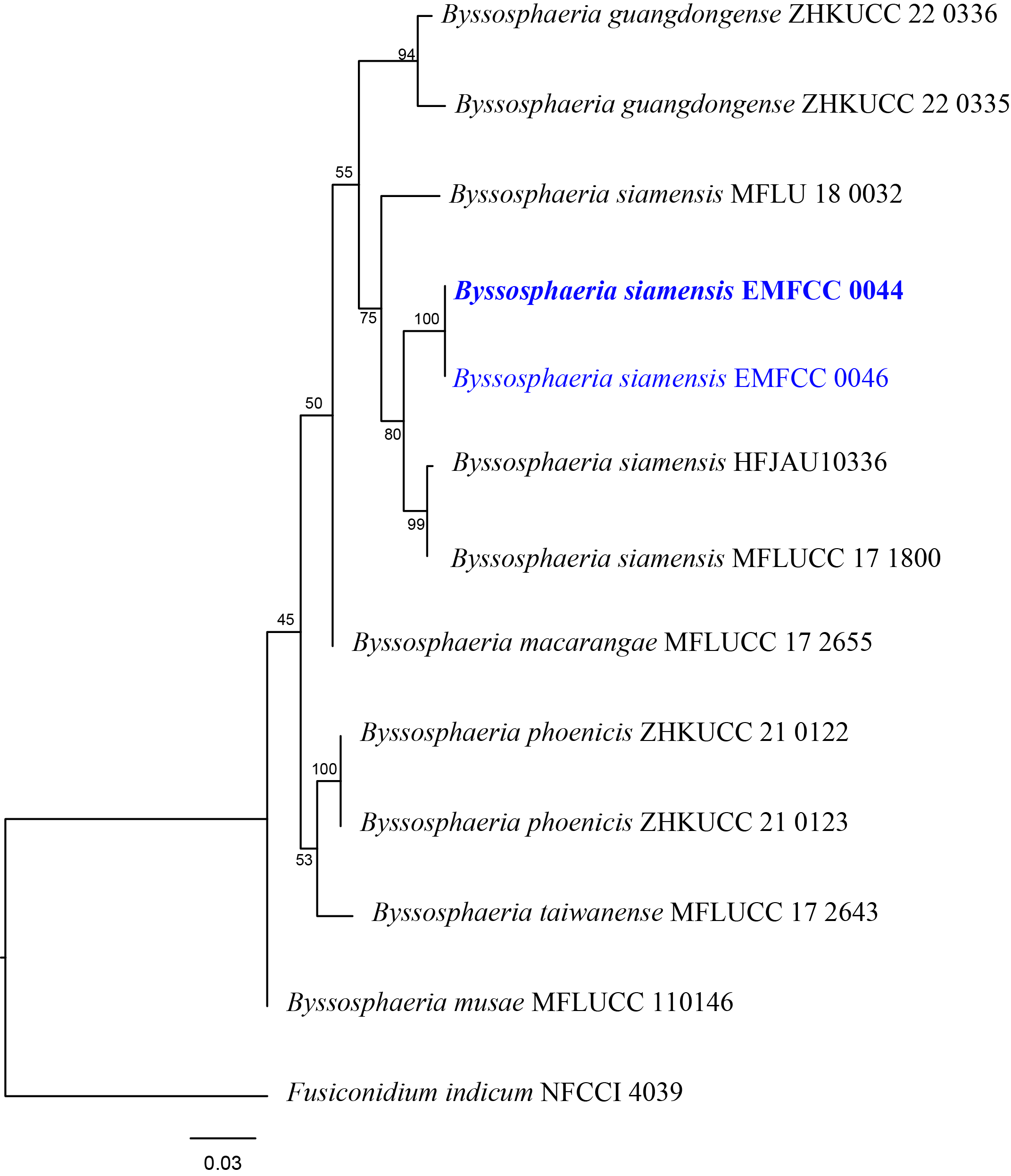


**Figure S8:** The best-scoring RAxML tree based on a concatenated ITS dataset of Byssosphaeria.


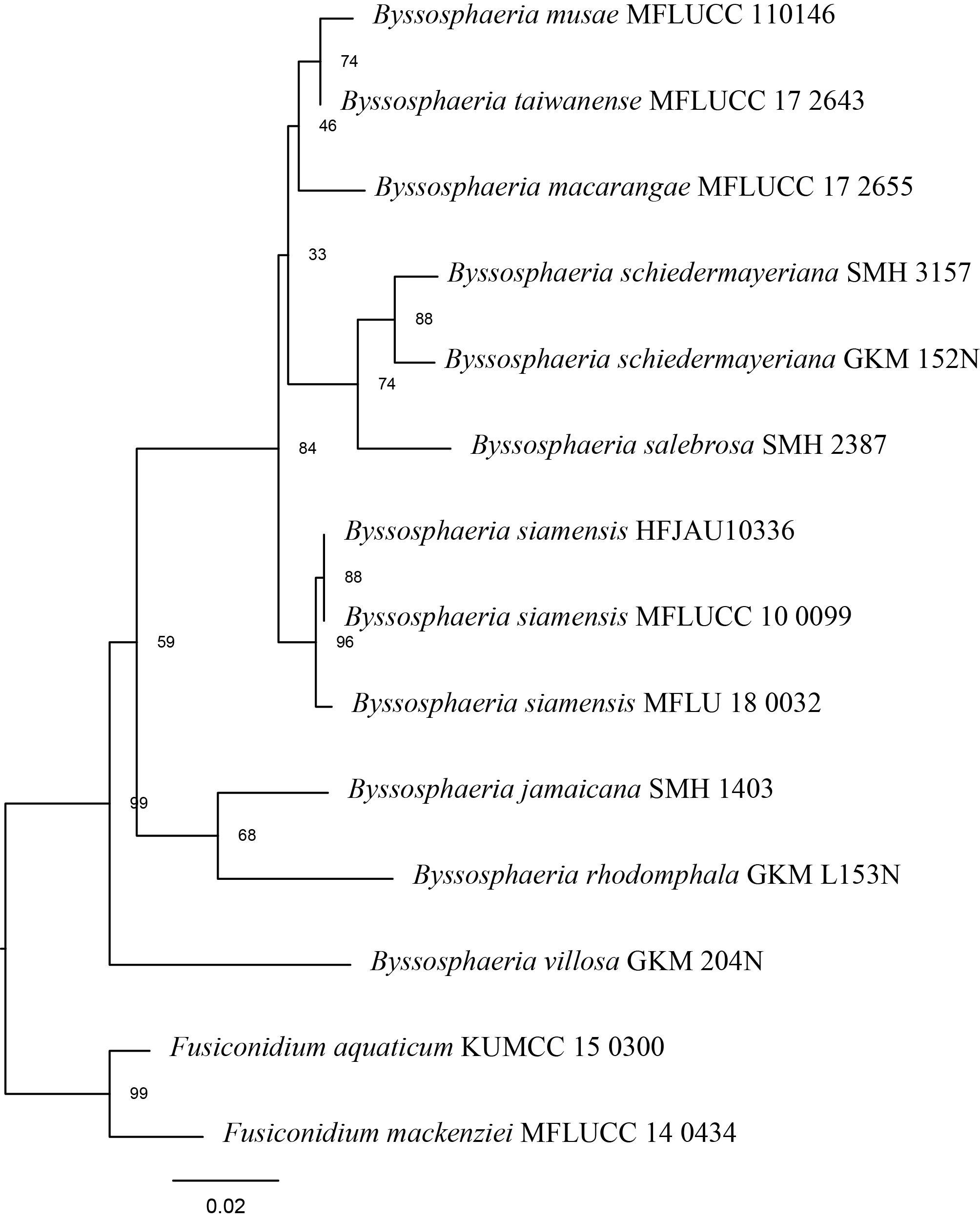


**Figure S9:** Phylogram generated from maximum parsimony analysis based on combined tef1-α dataset of Byssosphaeria.
